# From pores to rupture: Structural basis and regulation of lytic cell death by gasdermins and NINJ1

**DOI:** 10.1016/j.jbc.2025.110698

**Published:** 2025-09-12

**Authors:** Chengliang Wang, Brooke Dreyer, Evelyn Teran, Jianbin Ruan

**Affiliations:** Department of Immunology, University of Connecticut School of Medicine, UConn Health, Farmington, Connecticut, USA

**Keywords:** pyroptosis, necrosis, membrane rupture, gasdermin, GSDM, NINJ1, Ninjurin 1

## Abstract

Gasdermins (GSDMs) are a family of pore-forming proteins that execute lytic cell death by forming large β-barrel pores in cellular membranes. While traditionally regarded as the terminal effectors of pyroptosis, recent advances have revealed that GSDM pores alone are insufficient to cause full plasma membrane rupture, prompting the identification of nerve injury–induced protein 1 (Ninjurin 1 or NINJ1) as a critical executor of terminal cell lysis. This review provides an in-depth overview of the structural basis of GSDM pore formation and the regulatory mechanisms that govern their activity, including diverse post-translational modifications, such as ubiquitination, palmitoylation, and poly(ADP-ribosyl)ation. We also expand our discussion to the noncanonical activation strategies observed in bacterial, fungal, and ancient eukaryotic GSDM homologs. We further explore the molecular mechanisms for NINJ1 activation, highlighting its global role in mediating plasma membrane rupture downstream of multiple lytic cell death pathways. Finally, we discuss the pathological implications of dysregulated NINJ1 activity in related diseases, emphasizing its therapeutic potential as a universal modulator of terminal cell rupture.

Regulated cell death, often referred to as programmed cell death, is essential for maintaining tissue integrity, immune defense, and overall organismal health (1, 2). In multicellular organisms, the continual generation of new cells during development, immune responses, and tissue turnover necessitates a robust system for eliminating damaged, infected, or senescent cells (1, 3). Regulated cell death ensures this critical balance by orchestrating cellular removal in a controlled manner. Broadly, these pathways are categorized into two major forms: nonlytic and lytic cell death (4, 5).

Nonlytic cell death, typified by apoptosis, is immunologically silent (6, 7, 8). During apoptosis, the plasma membrane remains intact as cellular contents of the dying cell are neatly packaged in apoptotic bodies, which are efficiently cleared by phagocytes without triggering inflammation (8, 9, 10). In contrast, lytic cell death pathways—like pyroptosis and necroptosis—are defined by membrane rupture and the uncontrolled release of intracellular contents (11, 12, 13, 14, 15, 16, 17, 18). Despite differences in their signaling transductions, these lytic cell death pathways ultimately propagate inflammation, which is central to host defense yet implicated in pathological inflammation (11, 16, 18, 19, 20, 21, 22, 23).

Pyroptosis is currently the most well-characterized lytic cell death pathway. Though initially defined as a proinflammatory form of cell death mediated by caspase-1, the discovery of gasdermin D (GSDMD) and its pore-forming activity redefined pyroptosis as GSDM-mediated, programmed necrotic cell death (20, 24, 25, 26, 27). Six GSDM genes have been identified in humans, and GSDM homologs are found in chordates, mollusks, and even bacteria—underscoring the evolutionary importance of GSDMs (28, 29, 30, 31, 32, 33, 34). Among these, GSDMD is the most extensively studied, and much of what is currently understood about the GSDM family is extrapolated from its activation mechanism. Canonically, GSDMs are activated through proteolytic cleavage at their interdomain linker, which connects the autoinhibitory C-terminal domain (CTD) and the pore-forming N-terminal domain (NTD) (35, 36, 37, 38). Upon cleavage, the liberated NTD oligomerizes within membrane bilayers to form transmembrane (TM) pores that mediate the secretion of mature interleukin (IL)-1 family cytokines, including IL-1β and IL-18, and other protein or nonprotein cellular contents (35, 36, 39) (Fig. 1). Though initially thought to be sufficient for pyroptosis, GSDMD pore formation alone is insufficient to cause complete plasma membrane rupture—a critical event for the release of high–molecular-weight damage-associated molecular patterns (DAMPs) such as HMGB1 and the cytosolic enzymes like lactate dehydrogenase (LDH) (40, 41). This terminal step of pyroptosis requires the activity of a distinct membrane protein, nerve injury–induced protein 1 (Ninjurin 1 or NINJ1) (42). Recent studies demonstrate that NINJ1 oligomerizes on the plasma membrane to execute membrane rupture, acting downstream of GSDMD activation and functioning as a critical determinant of LDH and other DAMP release in pyroptosis (42, 43) (Fig. 1). Given the essential role of GSDM family proteins and NINJ1 protein in executing lytic cell death, accumulating structural studies have been conducted to unravel their mechanisms of activation, membrane engagement, and regulation.

**Figure 1:**
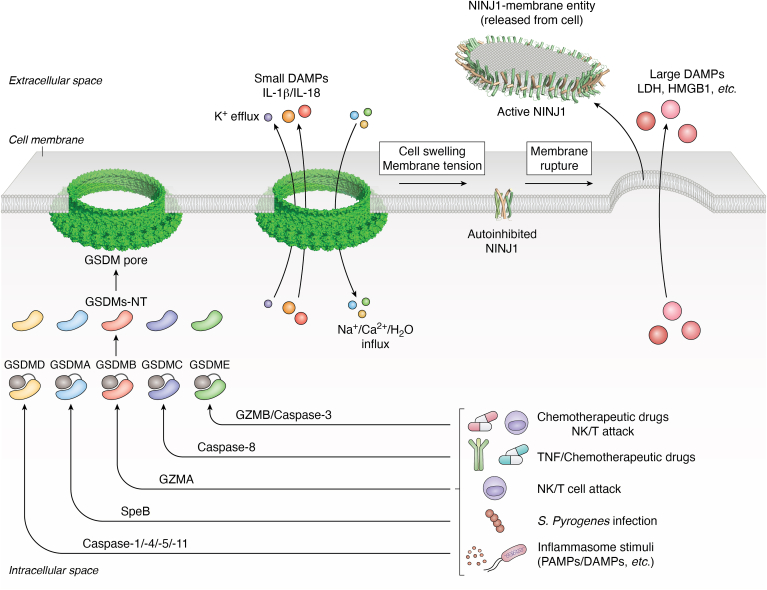
**Overview of gasdermin (GSDM)-mediated pyroptosis and NINJ1-mediated plasma membrane rupture.** The schematic illustrates the signaling cascade of GSDM-mediated pyroptosis and the subsequent terminal membrane rupture executed by NINJ1. In the canonical inflammasome pathway, pattern recognition receptors (PRRs) assemble inflammasomes to activate caspase-1, which cleaves GSDMD at its interdomain linker, liberating the N-terminal domain (GSDMD-NT) from the autoinhibitory full-length (GSDMD-FL) form. In the noncanonical pathway, intracellular lipopolysaccharide (LPS) directly activates caspase-4/-5 (human) or caspase-11 (mouse), which in turn cleaves GSDMD. The liberated GSDMD-NT translocates to the plasma membrane, oligomerizes, and inserts into lipid bilayers to form large β-barrel pores, permitting the release of small damage-associated molecular patterns (DAMPs) and proinflammatory cytokines, such as interleukins, IL-1β and IL-18, while also initiating ionic fluxes, including K^+^ efflux and Na^+^–Ca^2+^ influx, leading to osmotic imbalance and cell swelling. In addition to GSDMD, other GSDM family members are activated by distinct mechanisms: GSDMA is cleaved by bacterial virulence factor SpeB during *Streptococcus* infection; GSDMB is activated by granzyme A (GZMA) from cytotoxic lymphocytes; GSDMC is cleaved by caspase-8 upon TNF-α signaling or α-ketoglutarate-induced oxidative stress; GSDME is activated by caspase-3 downstream of apoptosis or by granzyme B (GZMB) from immune effector cells. While GSDM pores disrupt ionic homeostasis and initiate early membrane permeabilization, they are insufficient for releasing large cytoplasmic contents, such as HMGB1 and LDH. The terminal plasma membrane rupture step requires the activation of NINJ1. In its resting state, NINJ1 exists in an autoinhibited conformation as dimers or trimers. Upon osmotic swelling and membrane tension, NINJ1 undergoes conformational rearrangement to assemble into filamentous arrays, which excise portions of the plasma membrane, leading to catastrophic rupture. This sequential execution of pore formation by GSDMs followed by NINJ1-mediated rupture ensures the full release of large DAMPs and amplifies inflammatory signaling. LDH, lactate dehydrogenase; NINJ1 (Ninjurin 1), nerve injury–induced protein 1; TNF-α, tumor necrosis factor-alpha.

The recognition of GSDMs as pore-forming executioners of pyroptosis occurred approximately a decade ago, and the discovery of NINJ1 as the terminal effector of plasma membrane rupture is even more recent. These breakthroughs have rapidly reshaped our understanding of lytic cell death; however, given the nascent stage of this field, many critical questions remain unresolved. The precise mechanisms governing GSDM activation beyond canonical proteolytic cleavage, the triggers and regulation of NINJ1 oligomerization, and the broader implications of these pathways in immune responses, tissue homeostasis, and disease pathogenesis are areas of active and ongoing investigation. This review aims to synthesize current structural insights while highlighting these knowledge gaps and emerging directions in the field.

## GSDM pore formation and regulation

### The GSDM family and its evolution

The significance of the GSDM family came into sharp focus in 2015, when GSDMD was independently identified by two research groups as the critical substrate of inflammatory caspases and the direct executioner of pyroptotic cell death (24, 25). Using CRISPR–Cas9 genetic screens and biochemical analyses, these landmark studies revealed that caspase-1, -4, -5, and -11 cleave GSDMD at a conserved interdomain linker to release a pyroptotic active N-terminal fragment (24, 25, 44, 45). This discovery redefined the concept of pyroptosis from being a caspase-dependent process to one executed by a structurally conserved family of pore-forming proteins (27, 46). Since then, the GSDM family has emerged as a key component in innate immunity, inflammation, and cell death, with implications in infectious disease, cancer, and autoimmune disorders (47, 48, 49, 50).

**Figure 2:**
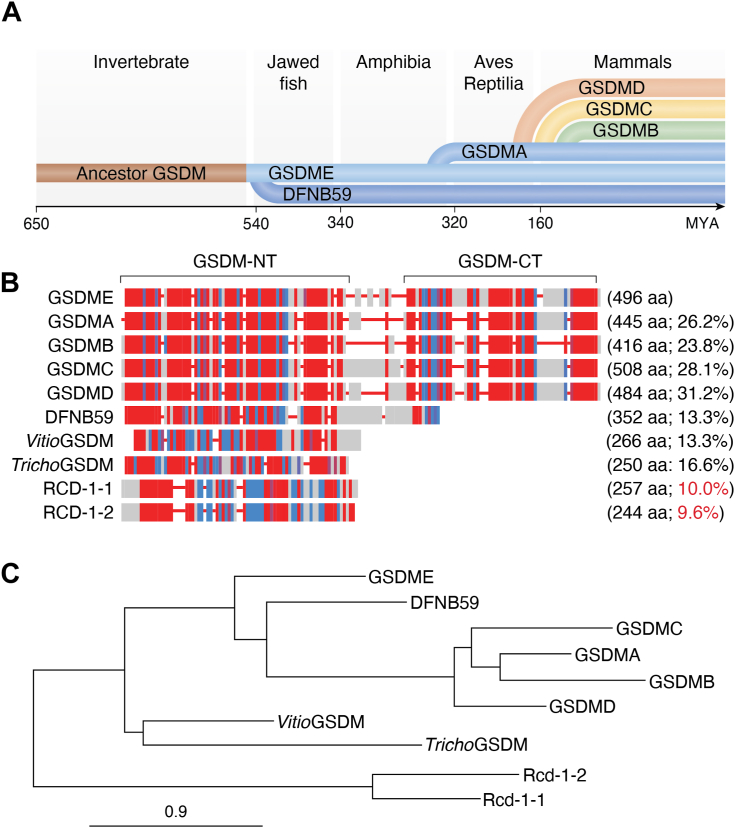
**Evolutionary analysis of the gasdermin (GSDM) family across species.***A*, evolutionary timeline of the GSDM gene family. Phylogenetic branching events are indicated along a timeline in million years ago (MYA), showing the origin of ancestral GSDM and subsequent gene duplication events leading to the emergence of GSDME, DFNB59, and lineage-specific expansions of GSDMA, GSDMB, GSDMC, and GSDMD in vertebrates. *B*, multiple sequence alignment of GSDM family members from vertebrates, bacteria, basal metazoans, and fungi. The alignment compares representative sequences from human GSDMs (GSDMA, GSDMB, GSDMC, GSDMD, GSDME, and DFNB59), bacteria *Vitiosangium* sp. GSDM, basal metazoan *Trichoplax adhaerens* GSDM, and fungi *Neurospora crassa* RCD-1-1 and RCD-1-2. Sequence identities are indicated. Conservation scores are color coded, with *red* indicating highly conserved residues and *blue* indicating less conserved ones. *C*, phylogenetic tree of GSDM family proteins. The unrooted phylogenetic tree was constructed based on full-length amino acid sequences, showing the evolutionary relationships among GSDMs from different species.

The GSDM family consists of GSDMA, GSDMB, GSDMC, GSDMD, GSDME (also known as DFNA5), and DFNB59 (also known as PJVK) in humans (28, 32). Each GSDM member possesses a conserved two-domain architecture: an NTD responsible for pore formation and a CTD that maintains autoinhibition under resting conditions (36). Evolutionary analyses reveal that GSDMs are ancient proteins, with GSDME being the most ancestral member, originating over 500 million years ago (32, 51) (Fig. 2*A*). Throughout evolution, this ancestral proto-GSDM gene underwent several duplication events leading to its diversification into distinct subfamilies, each with specialized functions (32). The first major diversification occurred approximately 475 million years ago, before the divergence of cartilaginous and bony fish, producing DFNB59 (Fig. 2*A*) (52). DFNB59 lost three exons encoding the C-terminal autoinhibitory domain, likely because of the incomplete duplication, resulting in a considerably shorter C terminus (Fig. 2*B*). Whether DFNB59 retains pyroptotic activity, and if so, how its truncated C terminus maintains the inactive state remains an open question. Another wave of gene expansion occurred around 320 million years ago, before the divergence of reptiles, turtles, and birds, leading to the emergence of GSDMA from a duplication of GSDME (51, 53). GSDMA subsequently serves as a mammalian GSDM ancestor for further lineage-specific duplications, giving rise to GSDMB, GSDMC, and GSDMD (Fig. 2*A*) (54). These paralogs ultimately acquired distinct regulatory and functional properties, contributing to the diverse mechanisms of cell death and host defense employed by modern vertebrates.

Interestingly, GSDM-like genes have been identified in bacteria and fungi and have been reported to induce cell lysis by membrane binding (31, 34, 55). However, these GSDMs—particularly the fungal GSDMs—share no or very low relevant sequence identity with invertebrate and vertebrate GSDMs (Fig. 2, *B* and *C*), suggesting these gene families are evolutionarily distinct analogs.

### Divergence in function and activation

Despite their conserved domain architecture, GSDMs exhibit remarkable functional diversity, shaped by distinct tissue distributions, activation mechanisms, and physiological roles (Table 1).

**Table 1:** The GSDM family

Human GSDMs	Mouse orthologs	Physiological tissue expression	Proteolytic activation (cleavage site)	Biological functions	Related diseases	PTMs	References
GSDMA	GSDMA 1-3	Gastrointestinal tract, skin epithelium	*Streptococcus pyogenes* SpeB (Gln246); Caspase-1 (nonmammals) (tetrapeptide motif YVAD or FASD)	Pyroptosis, restricting bacterial replication, facilitating immune clearance	Gastric cancer, systemic sclerosis	Phosphorylation of Thr8 by PLK1 inhibits pyroptotic activity; phosphorylation of Ser353 by Unc-51-like autophagy-activating kinase 1 leads to activation	(28, 54, 63, 64, 102, 105)
GSDMB (isoforms 1-6)	None	Airway epithelium, gastrointestinal tract, brain, endocrine tissue, bone marrow tissue, lymphocytes, *etc*.	Caspase-1 (Asp236); granzyme A (Lys244)	Pyroptosis, epithelial immunity and inflammation, antitumor immunity	Asthma, breast cancer, gastric cancer, cervical carcinoma, bladder cancer, inflammatory bowel disease	Ubiquitination by IpaH7.8 mediates 26S proteosome degradation	(65, 66, 67, 74, 75, 76, 97)
GSDMC	GSDMC 1-4	Cerebral cortex, endocrine tissues, skin, trachea, spleen, esophagus, stomach, intestines, vagina, bladder	Caspase-8 (Asp365 and Asp240)	Pyroptosis, hypoxia, inflammation, metabolic stress, antitumor immunity	Colorectal cancer, lung cancer, hepatocellular carcinoma	Unknown	(47, 77, 78, 79, 80)
GSDMD	GSDMD	Immune cells, gastrointestinal tract, placenta, endothelial cells	Caspase-1/-4/-5/-11 (Asp275 in humans or Asp276 in mice); caspase-8; neutrophil elastase (Val251); cathepsin G (Leu274)	Pyroptosis, immunity and inflammation	Sepsis, atherosclerosis, myocardial ischemia–reperfusion injury, acute kidney injury, acute lung injury, autoimmune diseases, infectious diseases, *etc.*	Itaconation of Cys77 and succination of Cys191 inhibit pyroptotic activity; phosphorylation of Ser46 by AMP-activated protein kinase and Thr213 by protein phosphatase 1 inhibit pyroptotic activity; S-palmitoylation of Cys191 enhances pyroptotic activity; ubiquitination by TRAF1 or SYVN1 enhances pyroptotic activity, and ubiquitination by IpaH7.8 leads to degradation	(24, 25, 26, 27, 38, 56, 57, 58, 59, 60, 61, 62, 93, 100, 101, 104, 106, 107, 108, 109)
GSDME (DFNA5)	GSDME (DFNA5)	Gastrointestinal tract, brain, uterus, smooth muscle cells	Caspase-3 (Asp270); granzyme B (Asp270)	Pyroptosis, antitumor immunity	Gastric cancer, colorectal cancer, breast cancer, hearing loss	Phosphorylation of Thr6 inhibits pyroptotic activity, palmitoylation of Cys407 and Cys408 enhances pyroptotic activity, PARylation of Asp229 and Glu233 enables activation	(81, 82, 83, 84, 85, 86, 93, 98, 125)
GSDMF (DFNB59)	GSDMF (DFNB59)	Auditory system	Unknown	Unknown	Autosomal recessive nonsyndromic hearing loss	Unknown	(87, 88, 89)

#### Gasdermin D

GSDMD is the most extensively characterized member of the GSDM family and serves as the central executor of pyroptosis in innate immune cells. GSDMD is broadly expressed in both immune and nonimmune cells, where it mediates inflammatory cell death and the release of cytokines and other DAMPs (56, 57). While GSDMD is primarily cleaved and activated by inflammatory caspases, including caspase-1, caspase-4/-5 in humans, and caspase-11 in mice, it is also activated through alternative pathways (24, 25, 26, 38). Caspase-8, which is traditionally associated with apoptotic signaling, can cleave GSDMD during specific death receptor or Toll-like receptor stimulations, particularly in the absence of caspase-1 (58, 59). In addition, neutrophil serine proteases, including neutrophil elastase and cathepsin G, can cleave mouse GSDMD at Val251 and Leu274 in activated neutrophils, linking GSDMD-dependent pyroptosis to neutrophil extracellular trap formation and inflammatory responses during infection (60, 61, 62). These diverse activation pathways highlight the central role of GSDMD as a versatile executor of lytic cell death across multiple immune contexts, integrating signals from inflammasomes, apoptotic pathways, and neutrophil activation to amplify host defense and inflammation.

#### Gasdermin A

GSDMA is exclusively expressed in the upper gastrointestinal tract and skin epithelium (28). Interestingly, the physiological function of GSDMA has diverged significantly between mammals and nonmammals. In many nonmammalian vertebrates, such as birds, amphibians, and reptiles, GSDMA contains conserved caspase-1 recognition motifs, such as YVAD or FASD, within the interdomain linker (54). These motifs allow GSDMA to be directly cleaved and activated by host-encoded caspase-1, functioning as a *bona fide* effector downstream of inflammasome signaling (54). In contrast, this caspase-1 cleavage site is disrupted in mammalian GSDMA, leading to a functional shift away from the original inflammasome signaling (54). As a result, mammalian GSDMA adopts a distinct immune surveillance role, acting as a sensor of microbial virulence factors. During *Streptococcus pyogenes* infection, the secreted bacterial cysteine protease SpeB specifically cleaves mouse and human GSDMA at Gln246, releasing the pore-forming active NTD (63, 64). This activation triggers pyroptotic cell death in keratinocytes, thereby restricting bacterial replication and facilitating immune clearance.

#### Gasdermin B

GSDMB is highly expressed in epithelial tissues, particularly those lining mucosal surfaces, such as the gastrointestinal and respiratory tracts, where it contributes to epithelial immunity and inflammation (65, 66, 67). Unlike other GSDM family members, GSDMB lacks a murine ortholog, and in humans, GSDMB exists in multiple isoforms generated through alternative splicing (68, 69). These isoforms differ in their interdomain linker length and sequence, which in turn influence their cleavage susceptibility and pyroptotic activity (70, 71, 72, 73). GSDMB was initially identified as a substrate of granzyme A (GZMA), a serine protease secreted by cytotoxic lymphocytes during immune-mediated killing. GZMA cleaves GSDMB primarily at Lys244, liberating the NTD to induce pyroptotic cell death in epithelial and cancer cells (74). In addition to GZMA, a recent study demonstrated that the longest isoform of GSDMB can be activated by caspase-1 at Asp236, suggesting a potential link between GSDMB and inflammasome signaling (75). However, the physiological significance of this cleavage remains to be fully elucidated. GSDMB is frequently upregulated in various cancers, including breast, gastric, and cervical carcinomas. Enhancing GSDMB-driven pyroptosis in tumor cells has been proposed as a strategy to potentiate immune-mediated tumor suppression (74, 76).

#### Gasdermin C

GSDMC exhibits a distinct expression profile. Although expressed in various normal tissues, GSDMC is markedly upregulated under pathological conditions, particularly in the tumor microenvironment (47, 77, 78). Hypoxia, inflammation, and metabolic stress have been implicated in the transcriptional induction of GSDMC, notably through the PD-L1–STAT3 signaling axis, which becomes activated in response to chemotherapeutic drugs (79). GSDMC is proteolytically activated through two caspase-8-dependent pathways involving tumor necrosis factor-alpha (TNF-α) and the metabolite α-ketoglutarate (79, 80). In the TNF-α pathway, caspase-8 cleaves GSDMC at Asp365, releasing a noncanonical pore-forming N-terminal fragment and inducing pyroptosis (79). In contrast, α-ketoglutarate treatment elevates intracellular reactive oxygen species (ROS), leading to the oxidation and endocytosis of death receptor 6 (80). Internalized death receptor 6 recruits pro-caspase-8 and GSDMC to form a receptosome complex, where GSDMC is cleaved at Asp240, triggering pyroptosis (80). These pathways highlight a context-dependent mechanism of GSDMC cleavage, in which the site and outcome of cleavage are determined by the upstream stimulus.

#### Gasdermin E

GSDME is broadly expressed across various human tissues, including the gastrointestinal tract, brain, uterus, and smooth muscle cells, and plays a pivotal role in converting apoptotic signals into pyroptotic cell death (81, 82). The expression of GSDME in tumors is often downregulated by promoter hypermethylation (83, 84), a mechanism that allows cancer cells to evade pyroptosis and resist immune surveillance. Restoration of GSDME expression in these contexts can sensitize tumor cells to inflammatory death (85). GSDME is primarily activated by caspase-3. Upon apoptotic stimuli, including chemotherapy or death receptor signaling, caspase-3 cleaves GSDME at a conserved Asp270 residue to trigger pyroptosis (85). In addition to caspase-3, granzyme B secreted by natural killer cells and cytotoxic T lymphocytes has also been shown to directly cleave and activate GSDME. Granzyme B cleaves GSDME at the same cleavage site as caspase-3 (86). This cleavage enables immune effector cells to directly induce GSDME-mediated pyroptosis in target cells, thereby enhancing immunogenic cell death and stimulating robust antitumor immune responses (86).

#### DFNB59

DFNB59 is predominantly expressed in the auditory system and plays a critical role in maintaining the function and integrity of auditory pathway neurons (87, 88). Loss-of-function mutations in DFNB59 are linked to autosomal recessive nonsyndromic hearing loss, specifically auditory neuropathy, a condition characterized by impaired neural transmission of sound despite intact cochlear hair cell function (88). DFNB59 is unique in the GSDM family because of its truncated CTD. Although DFNB59 retains conserved lipid-binding interfaces within its NTD, a recent study has proposed it as a nonpyroptotic GSDM (32, 89). However, given that pore assembly and membrane lytic activity in GSDMs are tightly regulated by a range of context-dependent mechanisms, such as post-translational modifications (PTMs), the functional potential of DFNB59 requires in-depth investigation.

### Structural transition upon GSDM pore formation

**Figure 3:**
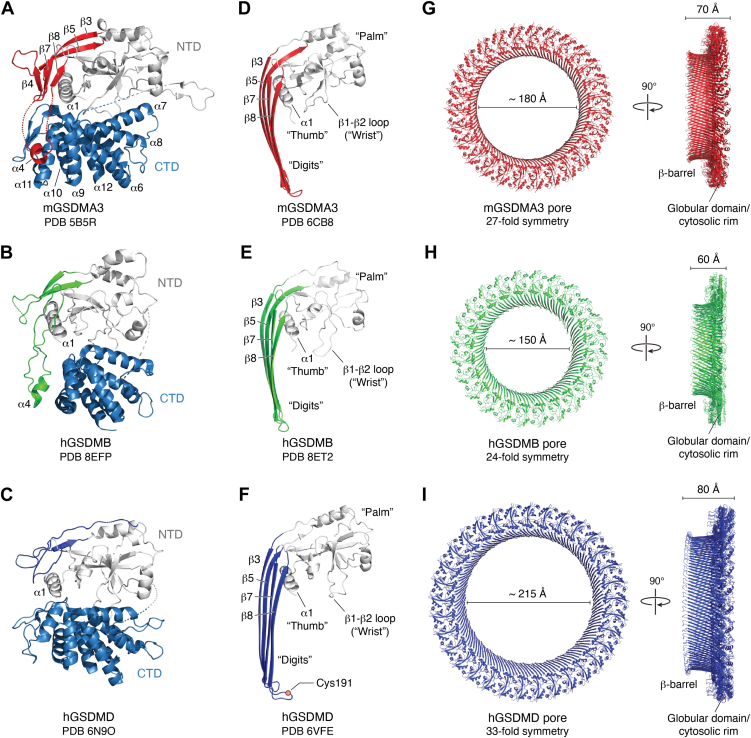
**Structural transitions of mammalian gasdermins (GSDMs) upon pore formation.***A*–*C*, structures of mouse GSDMA3 (mGSDMA3), human GSDMB (hGSDMB), and human GSDMD (hGSDMD) in their autoinhibited, full-length conformations. The N-terminal domain (NTD), globular subdomain (*gray*), transmembrane β-hairpins (*red*), and key structural elements (α1 helix and β1–β2 loop) are labeled. Structures: mGSDMA3 (Protein Data Bank [PDB] code: 5B5R), hGSDMB (PDB code: 8EFP), hGSDMD (PDB code: 6N9O). *D*–*F*, conformational rearrangements upon membrane association and pore formation. The NTD undergoes dramatic reorganization, with β3, β5, β7, and β8 forming extended β-strands (“digits”), whereas the globular domain serves as a “palm.” The α1 helix acts as a “thumb,” and the β1–β2 loop anchors membrane binding. Structures: mGSDMA3 (PDB code: 6CB8), hGSDMB (PDB code: 8ET2), and hGSDMD (PDB code: 6VFE). *G*–*I*, top and side views of reconstructed GSDM pores, illustrating their oligomeric assemblies and inner diameters. Mouse GSDMA3 forms 24- to 28-fold symmetric pores (inner diameter ∼180 Å), human GSDMB forms 24- to 26-fold symmetric pores (∼150 Å), and human GSDMD assembles into 31- to 34-fold symmetric pores. The overall architecture is conserved, but oligomeric stoichiometries and pore sizes vary among family members.

As mentioned above, GSDMs consist of a pore-forming NTD and an autoinhibitory CTD connected by a flexible interdomain linker (36, 90). The NTD comprises a globular subdomain, which features a twisted β-sheet core surrounded by several α-helices, and a largely disordered TM region enriched in hydrophobic residues responsible for membrane insertion. The CTD adopts a globin-like α-helical fold, consisting of nine helices capped by a short antiparallel three-stranded β-sheet. In the autoinhibited full-length conformation, the CTD tightly interacts with the NTD through two major interfaces, effectively concealing the lipid-binding and membrane insertion motifs (36, 37, 90, 91). In interface I, helices α5, α7, α8, and α12 of the CTD form a large hydrophobic pocket to accommodate the lipid-binding α1 helix and β1–β2 hairpin of the NTD. While in interface II, CTD helices α9 and α11 engage extensively with the α4 helix projecting from the NTD (Fig. 3, *A*–*C*). These extensive interactions sequester the lipid-binding interfaces and stabilize the flexible and hydrophobic TM subdomain within NTD, thereby maintaining the full-length GSDMs as inert, soluble cytosolic proteins in their resting state (36, 90).

While proteolytic cleavage between the NTD and CTD has long been recognized as the canonical activation mechanism for most mammalian GSDMs, accumulating evidence indicates that this may not be a universal feature. Recent studies have uncovered alternative activation strategies, including cleavage-independent mechanisms—as observed in ancient fungal GSDMs (92)—and regulatory PTMs, such as palmitoylation (93), that can modulate GSDM activation. Despite these diverse activation pathways, the structural transition upon activation—namely, the reorganization of the NTD into an amphipathic, membrane-inserting β-barrel pore—is remarkably conserved across species and GSDM homologs. Typically, the NTD translocates to plasma or organelle membranes upon release from CTD (35, 36, 38, 94). Once associated with membranes, the NTD undergoes dramatic conformational changes within the TM subdomain. The disordered β3–β4–β5 and β7–β8 regions are structurally reorganized into two extended TM β-hairpins stabilized by the hydrophobic membrane environment (Fig. 3, *D*–*F*). As a result, the pore-forming GSDM-NTD protomer adopts a left-handed conformation, consisting of a globular subdomain as the “palm,” the lipid-binding α1 helix and β1–β2 hairpin as the “thumb” and “wrist,” respectively, and the four long β-strands in the TM subdomain extending outward like “digits.” Interactions between both the TM β-strands and the globular subdomains drive the assembly of a large TM β-barrel pore (37, 39, 70, 71) (Fig. 3, *D*–*F*). *In vitro* reconstitution showed that the assembled GSDM pores typically consist of 24 to 34 NTD protomers with inner diameters ranging from 15 to 21.5 nm, sufficiently wide to allow the release of cytokines as well as small intracellular mediators, including galectins (Fig. 3, *G*–*I*). In addition, the conduits of the GSDM pores, such as those formed by GSDMD and GSDMA3, are predominantly negatively charged, making them preferentially facilitate the transport of the basic and neutral cargos (37, 39, 70, 71).

It should be noted that recent high-resolution atomic force microscopy (AFM) studies revealed that GSDM-NTD can assemble into diverse oligomeric structures beyond the classical ring-shaped pores (95, 96). Specifically, AFM imaging of mouse GSDMA3 and human GSDMD on supported lipid membranes identified the formation of arc- and slit-shaped oligomers. These noncircular assemblies are still capable of inserting into lipid bilayers and forming functional pores, indicating that a complete ring structure is not strictly required for membrane permeabilization. Time-lapse AFM further demonstrated that arc-shaped oligomers can elongate and progressively merge into slit-shaped or full-ring structures over time, suggesting a dynamic and flexible assembly process. This structural plasticity may enable GSDMs to function in varied membrane environments, potentially facilitating the release of cellular contents even when full ring formation is impeded (95, 96).

### Membrane attachment

GSDMs target membranes through selective recognition of acidic phospholipids, such as phosphatidylinositol phosphates, phosphatidic acid, phosphatidylserine, and cardiolipin. Phosphatidylinositol phosphates, phosphatidic acid, and phosphatidylserine are components of the inner leaflet of the plasma membrane, indicating that the proteolytically released GSDM-NTD can only kill the cell intracellularly (35). While cardiolipins are predominantly found in mitochondrial and bacterial membranes, this suggests a potential role for the GSDM pore in targeting intracellular bacteria or mitochondria during immune responses (35, 36, 97).

The lipid binding of GSDM-NTD is primarily mediated by the N-terminal α1 helix and the β1–β2 loop, where clusters of basic residues facilitate electrostatic interactions with acidic phospholipids, whereas the hydrophobic tip of the β1–β2 loop inserts into the lipid bilayer as a membrane anchor (37, 39, 70, 71, 90) (Fig. 3, *D*–*F*). A less conserved basic lipid-binding site was also identified in GSDMD and GSDMB, contributed by residues in the β7 and β8 strands within the TM subdomain. Notably, two recent structural studies of GSDMB revealed that the interdomain linker region is also directly involved in lipid binding (70, 71). This linker region varies among GSDMB isoforms because of alternative splicing, highlighting its regulatory role in modulating lipid-binding affinity and pore-forming activity (70, 71, 72, 73). Importantly, this additional lipid-binding site is conserved in GSDMD, and mutation of basic residues in this region significantly impairs its pyroptotic activity, underscoring the critical role of the interdomain linker (71). Although alternative splicing of the interdomain linker has not been identified in other GSDM family members, it remains plausible that the pyroptotic activity of GSDMs could be modulated through other regulatory mechanisms targeting this region, such as PTMs (*e.g.*, phosphorylation, ubiquitination, or palmitoylation), which may alter its structural conformation, membrane affinity, or protease accessibility. Further investigation into this region may uncover conserved or context-specific strategies for tuning GSDM activity across cell types and stimuli.

### Regulation of GSDM-mediated pyroptosis

GSDMs are subject to intricate layers of regulation that fine-tune their pore-forming activity and ensure appropriate pyroptotic responses. These regulatory mechanisms include diverse PTMs, such as palmitoylation (93, 98, 99, 100, 101), phosphorylation (102, 103, 104, 105), succination (106), ubiquitination (97, 107, 108, 109, 110, 111), and oxidative modification (112, 113, 114), as well as mechanisms beyond chemical modifications, such as extracellular vesicle (EV)–mediated transfer of GSDM pores (115).

#### Palmitoylation

Palmitoylation is a reversible lipid modification in which a 16-carbon saturated fatty acid (palmitate) is covalently attached to a cysteine residue *via* a thioester bond (116). This modification increases the hydrophobicity of proteins, promotes their association with cellular membranes, regulates their localization and stability, and modulates protein–protein interactions (116). Recent studies have revealed that GSDMD can be palmitoylated by the palmitoyl acyltransferases ZDHHC5 and ZDHHC9, both of which are upregulated in response to inflammatory stimuli and elevated ROS levels (93, 100, 101). Palmitoylation occurs at residue Cys191 in human GSDMD (Cys192 in mouse), a residue located within the TM subdomain of NTD (93, 99, 100, 101, 117) (Fig. 3*F*). While this modification does not affect the efficiency of caspase-mediated cleavage, it may trigger a conformational transition in the TM region that facilitates membrane anchoring and insertion (93, 100, 101, 117). Notably, the small-molecule inhibitors, disulfiram, necrosulfonamide, and Bay 11-7082, all target this PTM by covalently modifying Cys191, thereby effectively inhibiting pyroptosis (118, 119). It is important to note that these compounds exert their inhibitory effects through nonspecific thiol-reactive chemistry. Their action is not exclusive to GSDMD but relies on broadly reactive electrophilic groups that can modify any accessible cysteine residue within proteins. Therefore, while these compounds provide valuable tools to probe GSDMD function, their promiscuous binding underscores the need for the development of more selective inhibitors that target GSDMs through defined structural interfaces rather than generic cysteine alkylation.

Both full-length GSDMD and cleaved GSDMD-NTD can undergo palmitoylation. In full-length GSDMD, palmitoylation reduces the inhibition imposed by the CTD, thereby sensitizing GSDMD to activation (93). Remarkably, palmitoylated full-length GSDMD can independently form pores and induce pyroptosis, suggesting that proteolytic cleavage is not the sole determinant of GSDMD activation and palmitoylation may act as an alternative or parallel activation mechanism, particularly under conditions of oxidative stress or in subcellular environments where active proteases are not available (93).

Beyond GSDMD, palmitoylation has also been detected on other GSDM family members, including GSDMA, GSDMB, GSDMC, and GSDME, among which GSDME exhibits modification at both NTD and CTD (93, 98, 100). Palmitoylation of the GSDME-CTD does not directly regulate pore-forming activity but facilitates the dissociation of the NTD from the CTD, thereby promoting activation (98). The functional significance of palmitoylation in other GSDMs is currently less well characterized; however, it is well recognized that this lipid modification likely regulates GSDM pyroptotic activity through a conserved mechanism by modulating protein conformation and membrane association. Given the differential expression of GSDMs across immune, epithelial, and tumor tissues, palmitoylation likely acts as a dynamic and context-dependent modulator of GSDM activity in response to infection, inflammation, or cellular stress.

#### Ubiquitination

Ubiquitination is another critical PTM that can either suppress or enhance the pyroptotic activity of GSDMs, depending on the nature of the ubiquitin linkage and the target residues (120). Cleaved GSDMD-NTD has been shown to undergo K63-linked polyubiquitination at Lys237 mediated by the E3 ubiquitin ligase TRAF1, as well as K27-linked polyubiquitination at Lys203 and Lys204 mediated by SYVN1 (109). Inhibition of these modifications, either through genetic silencing of the E3 ligases or mutagenesis of the ubiquitinated lysines, significantly attenuates GSDMD-driven membrane permeabilization and the secretion of inflammatory cytokines, such as IL-18 and TNF-α (109). Although these modifications are proposed to enhance GSDMD-NTD oligomerization and pore formation, the precise structural mechanisms underlying these processes remain to be fully elucidated. In contrast, some other E3 ligases can negatively regulate GSDMD-dependent pyroptosis. E3 ligase RING1 directly binds GSDMD and catalyzes K48-linked polyubiquitination at Lys51 and Lys168, targeting it for subsequent proteasomal degradation and thereby inhibiting pyroptotic cell death (110). Similarly, IpaH7.8, a pathogen-derived E3 ligase employed by *Shigella flexneri*, also directly antagonizes GSDM-mediated pyroptosis. IpaH7.8 hijacks host ubiquitin machinery and targets human GSDMD and GSDMB for K48-linked polyubiquitination, leading to their proteasomal degradation (97, 108). This allows *Shigella* to evade innate immune defenses and establish infection. Interestingly, mice lack a GSDMB ortholog, and murine GSDMD cannot be efficiently recognized by IpaH7.8 because of sequence divergence at key interaction sites (70, 71, 121). As a result, mice retain functional GSDM-mediated pyroptotic responses and exhibit greater resistance to *Shigella* infection (108, 122).

In addition, GSDME is also subject to K48-linked polyubiquitination, specifically at Lys120 and Lys189 (111). While the E3 ligase responsible for this modification remains unidentified, USP48 has been identified to be the key deubiquitinase removing these ubiquitin chains, thereby stabilizing GSDME and enhancing its pyroptotic potential (111). This regulation has significant implications in cancer biology, where GSDME-mediated pyroptosis plays a key role in stimulating antitumor immunity. For instance, infection with oncolytic parapoxvirus ovis was found to reduce GSDME ubiquitination, thereby promoting GSDME-dependent pyroptosis in tumor cells and contributing to therapeutic efficacy (123).

#### PARylation

PARylation, or poly(ADP-ribosyl)ation, which plays crucial roles in regulating DNA repair, chromatin structure, and cell death signaling (124), has recently emerged as a novel PTM regulating GSDM activity. A recent study demonstrated that intense DNA damage, such as that induced by ultraviolet-C irradiation, activates nuclear poly(ADP-ribose) polymerase 1, leading to the synthesis of poly(ADP-ribose) polymers (125). These polymers then translocate to the cytoplasm and activate poly(ADP-ribose) polymerase 5, which facilitates the PARylation of full-length GSDME at Asp229 and Glu233. Similar to palmitoylation, PARylation induces a conformational change in full-length GSDME that relieves its autoinhibition, enabling its pore-forming activity without the need for proteolytic cleavage (125). In parallel, ultraviolet-C-induced oxidative stress promotes cardiolipin peroxidation and accumulation of lipid ROS, which are sensed by PARylated GSDME to drive its oligomerization and membrane insertion, ultimately leading to pyroptotic cell death (125). To date, PARylation has not been observed in other GSDMs. Whether this modification represents a unique regulatory mechanism for GSDME or a broader paradigm applicable to other GSDMs remains an open question that requires further investigation.

#### Phosphorylation

Phosphorylation has long been recognized as a negative PTM that suppresses the pore-forming activity of GSDMs. For instance, GSDMA is phosphorylated at Thr8 by Polo-like kinase 1, GSDME at Thr6 by AMP-activated protein kinase, and GSDMD at Ser46 and Thr213 by AMP-activated protein kinase and protein phosphatase 1, respectively (102, 103, 104, 126, 127). All these phosphorylation sites are structurally positioned within lipid-binding interfaces—Thr8 in GSDMA, Thr6 in GSDME, and Ser46 in GSDMD, or at the oligomerization interface—Thr213 in GSDMD. Phosphorylation at these residues likely alters the local electrostatic landscape, introducing steric hindrance or charge repulsion that impairs either membrane binding or pore assembly. However, a recent study suggests that phosphorylation may also act as a trigger to activate GSDMs (105). It was shown that phosphorylation of Ser353 on human GSDMA-CTD by Unc-51-like autophagy-activating kinase 1 under nutrient stresses induces a GSDMA-dependent pyroptosis. Introducing a phosphomimetic mutation at Ser353 causes spontaneous activation of full-length GSDMA (105). It has to be mentioned that Ser353 is not located within or near the interdomain interfaces, which raises intriguing questions about the structural mechanism underlying this activation.

#### EV-mediated transplantation of GSDM pores

In addition to PTMs, a recent study uncovers a novel, non–cell-autonomous mechanism of pyroptosis propagation involving EVs that carry functional GSDM pores (115). It was shown that supernatants from pyroptotic cells could induce cell death in adjacent bystander cells without direct contact, implicating a soluble factor in this process. Further analysis identified EVs as the carriers of this effect and confirmed the presence of GSDMD pores on the surface of these EVs. When these EVs interact with recipient cells, the GSDMD pores integrate into the plasma membrane, compromise membrane integrity, and trigger secondary pyroptosis (115). Notably, this EV-mediated bystander killing is not limited to GSDMD but may extend to other GSDMs, indicating a broader mechanism of intercellular pyroptotic dissemination. Traditionally, GSDM-mediated pyroptosis is considered a cell-intrinsic process, confined to the cell in which it is initiated; however, this discovery reshapes the paradigm by demonstrating that GSDMs can exert cytotoxic effects extracellularly *via* vesicular transport (115, 128). So far, the physiological and pathological relevance of EV-mediated transplantation of pyroptosis remains unclear. Key open questions include how this process modulates immune cell dynamics and tissue microenvironments and whether it contributes to the progression of human diseases, such as sepsis, autoimmunity, and cancer. Furthermore, the precise mechanisms governing the incorporation of GSDMD pores into EVs and their subsequent integration into recipient cell membranes also need further investigation.

### Unique activation mechanisms in bacterial and ancient eukaryotic GSDMs

#### Mechanism of pore formation in bacterial GSDMs

**Figure 4:**
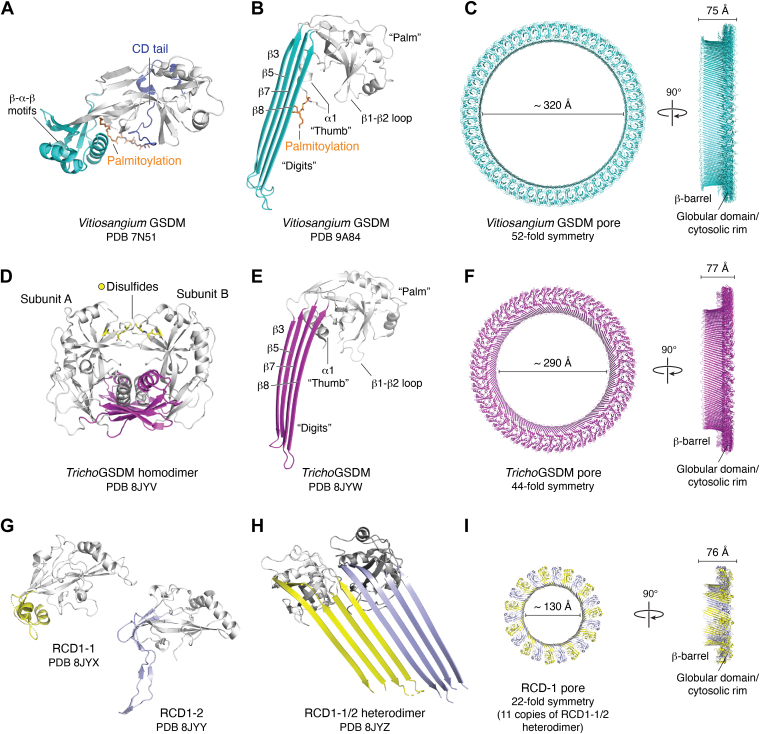
**Structural mechanisms of pore formation in bacterial and ancient eukaryotic gasdermins (GSDMs).***A*, crystal structure of bacteria *Vitiosangium* sp. GSDM (Protein Data Bank [PDB] code: 7N51) in the autoinhibited conformation. The palmitoylated N-terminal α-helix and the short C-terminal tail stabilize the inactive state by shielding lipid-binding interfaces. *B* and *C*, cryo-EM structure of *Vitiosangium* GSDM pore (PDB code: 9A84), forming a 52-fold symmetric β-barrel pore with an inner diameter of ∼320 Å. The activated *Vitiosangium* GSDM subunit adopts a highly conserved “hand-shaped” architecture composed of a globular domain (*palm*) and transmembrane β-strand “digits. *D*, crystal structure of *Trichoplax adhaerens* GSDM homodimer (PDB code: 8JYV) in the inactive conformation. The homodimer is stabilized through intermolecular disulfide bonds, sequestering lipid-binding regions. *E* and *F*, cryo-EM structure of the *Trichoplax* GSDM pore (PDB code: 8JYW), assembled into a 44-fold symmetric β-barrel with an inner diameter of ∼290 Å. *G*, crystal structures of *Neurospora crassa* RCD-1-1 (PDB code: 8JYX) and RCD-1-2 (PDB code: 8JYY) monomers, which remain inactive in the absence of allelic pairing. *H* and *I*, cryo-EM structure of the RCD-1 pore, composed of 22 subunits (11 RCD-1-1/2 heterodimers) arranged in a β-barrel with an inner diameter of ∼130 Å.

GSDM homologs have been identified across a wide range of species, including bacteria and fungi (30, 31). In bacteria, GSDM-like proteins (*b*GSDMs) are typically encoded within antiphage defense islands and are part of abortive infection systems that trigger bacterial death upon phage infection (31). This form of altruistic cell death protects the bacterial community by sacrificing infected bacteria to restrict viral replication and thereby protect the broader bacterial population. Despite their functional resemblance to mammalian GSDMs, *b*GSDMs exhibit notable structural differences with a remarkably truncated CTD ranging from a dozen to around 100 amino acids (31). Nonetheless, structural studies show that *b*GSDMs still retain an autoinhibited conformation similar to that of mammalian GSDMs (Fig. 4*A*). The short CTD peptide wraps around the lipid-binding interfaces of the NTD to block membrane engagement and stabilize the inactive state (31). Moreover, unlike mammalian GSDMs, in which the TM β-hairpin regions are disordered and require stabilization from CTD, *b*GSDMs adopt preformed β–α–β motifs in this region, providing intrinsic structural stabilization. In addition, a 16-carbon palmitate moiety from the palmitoylated cysteine in the N-terminal α helix further reinforces this inactive conformation by anchoring the hydrophobic elements in NTD (Fig. 4*A*). Notably, this cysteine is highly conserved across most bacterial and some fungal GSDMs (31).

Activation of *b*GSDMs also requires the proteolytic cleavage by associated proteases, such as caspase-like peptidases (31, 129). After cleavage, the short CTD peptide is released, liberating the pore-forming NTD. Upon activation, *b*GSDMs form ring-shaped β-barrel pores, similar to those formed by mammalian GSDMs, though typically larger, with *Bacteroidetes* pores composed of 30 to 31 subunits and *Vitiosangium* pores containing over 50 protomers (129). Despite their low sequence identity with mammalian GSDMs, *b*GSDMs preserve a conserved “hand-shaped” architecture in pore form (Fig. 4*B* and *C*). Molecular dynamics simulations further reveal a critical role of palmitoylation in *b*GSDM pore formation. The palmitate moiety, which initially contributes to maintaining the autoinhibited state in full-length *b*GSDMs, is repositioned to enhance the membrane affinity of *b*GSDM-NTD and facilitate the structural reorganization and membrane insertion of the β-hairpin TM region (31, 129). These mechanistic insights into palmitoylation-driven pore formation in *b*GSDMs may offer a valuable framework for understanding and manipulating palmitoylation in mammalian GSDMs.

#### Unique pore formation mechanisms in ancient eukaryotic GSDMs

While accumulating evidence has demonstrated a conserved mechanism for GSDM activation, whereby autoinhibited monomeric GSDMs are activated by proteolytic cleavage, recent studies have identified GSDMs in ancient eukaryotic organisms that utilize distinct autoinhibited and cleavage-independent activation mechanisms (30, 34, 92, 130).

*Tricho*GSDM, identified in the basal metazoan *Trichoplax adhaerens*, is a 250-residue protein consisting of a single domain homologous to the mammalian GSDM-NTD (92). Due to the lack of a CTD, *Tricho*GSDM has to form a homodimer to maintain the inactive state. In the *Tricho*GSDM homodimer, the two *Tricho*GSDM molecules are arranged in a symmetric “palm-to-palm” mode, in which the lipid-binding interfaces are buried within the dimer interface, thereby preventing membrane association. This homodimerization is further stabilized by intermolecular disulfide bonds formed between cysteines located in the lipid-binding β1–β2 loop (92) (Fig. 4*D*). Activation of *Tricho*GSDM is independent of proteolytic cleavage but relies on the reduction of disulfide bonds. Disulfide reduction triggers the dissociation of the *Tricho*GSDM homodimer, allowing the *Tricho*GSDM monomer to bind to the membrane and form pores (92) (Fig. 4, *E* and *F*).

In addition to *T. adhaerens*, recent studies have identified another two GSDM-NTD–only proteins, RCD-1-1 and RCD-1-2, in the filamentous fungus *Neurospora crassa* (30, 34, 55, 92). RCD-1-1 and RCD-1-2 are encoded by incompatible *rcd-1* alleles in different *N. crassa* strains and play a crucial role in allorecognition-induced programmed cell death (30). Structural studies reveal that both RCD-1 proteins adopt the canonical GSDM-NTD fold (92) (Fig. 4*G*). Interestingly, despite their ability to independently bind phospholipids and associate with membranes, each RCD-1 protein remains monomeric and lacks pore-forming activity when expressed alone. Activation of RCD-1 proteins requires allelic incompatibility, where RCD-1-1 and RCD-1-2 physically encounter each other and form functional heterodimers through specific intermolecular interfaces (92) (Fig. 4, *G* and *H*). Cryo-EM studies revealed that these heterodimers subsequently assemble into symmetric oligomeric pores composed of 11 alternating RCD-1-1 and RCD-1-2 subunits (Fig. 4*I*). Importantly, the oligomerization interfaces of RCD-1-1 and RCD-1-2 are structurally incompatible for homotypic interactions but complementary for heterotypic assembly (92). This ensures that only the copresence of both allelic variants leads to productive pore formation and cell death.

## NINJ1-mediated plasma membrane rupture

### Identification of NINJ1 as an executioner of plasma membrane rupture

While GSDMs have long been recognized as the central effectors of pyroptosis, emerging evidence has demonstrated that GSDM pore formation alone is insufficient to drive terminal plasma membrane rupture. GSDM pores allow ionic flux and early membrane permeabilization; however, under certain conditions, such as extracellular glycine treatment, where cells remain morphologically intact despite fully formed GSDM pores, the release of large cytoplasmic contents like LDH is inhibited (131, 132, 133, 134). This uncoupling between GSDM pore formation and plasma membrane rupture prompted a search for the missing executioner downstream of GSDMs, ultimately leading to the identification of NINJ1 as a critical mediator of terminal membrane rupture (42).

NINJ1 was originally identified in 1996 as a nerve injury–induced protein involved in nerve regeneration and cell adhesion (135). Although several studies have shown that NINJ1 is involved in the inflammatory response in different tissues, it was in 2021 that NINJ1 was identified as an essential gene mediating plasma membrane rupture through a forward-genetic screen using bone marrow–derived macrophages (BMDMs) from mice mutagenized with *N*-ethyl-*N*-nitrosourea (42). The study revealed that NINJ1-deficient BMDMs exhibited normal GSDMD pore formation and cytokine release but developed a distinctive and persistent ballooned morphology and were unable to release large intracellular proteins, such as HMGB1 and LDH, clearly demonstrating that NINJ1 operates downstream of GSDMD (42).

### Mechanism of NINJ1-mediated plasma membrane rupture

**Figure 5:**
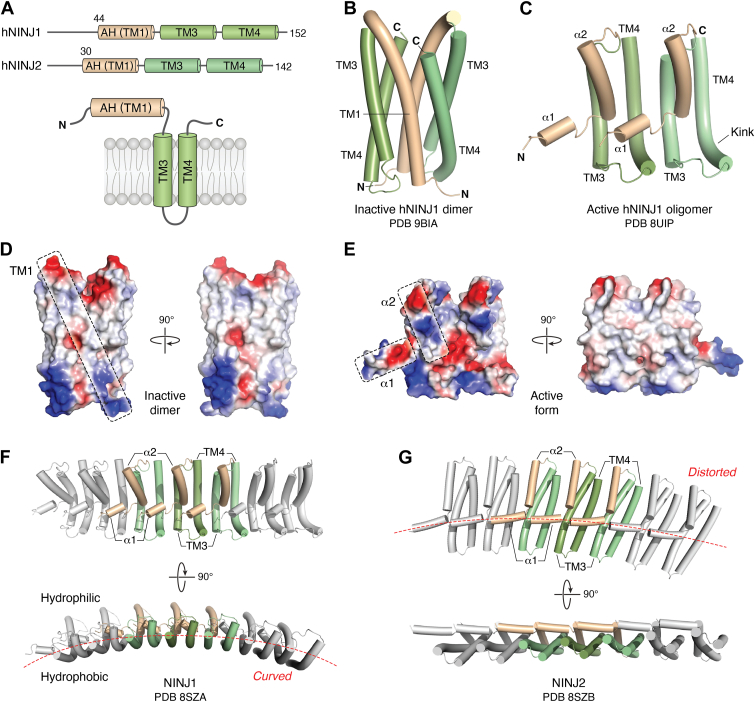
**Structural basis of NINJ1 activation and membrane rupture.***A*, domain architecture of human NINJ1 and NINJ2. Both proteins contain two predicted transmembrane (TM) helices (TM3 and TM4) and an extracellular N-terminal amphipathic helix (AH; TM1), which is repositioned during activation. Despite sharing 52% sequence identity, only NINJ1 exhibits membrane-lytic activity. The N-terminal regions—44 residues in NINJ1 and 30 in NINJ2—are disordered and of unknown function. *B*, cryo-EM structure of inactive NINJ1 homodimer (Potein Data Bank [PDB] code: 9BIA). Inactive NINJ1 forms a face-to-face dimer embedded in the plasma membrane. The N-terminal TM1 helix is repositioned into the membrane and packs against TM3 and TM4, forming a compact three-helix bundle stabilized by extensive intramolecular interactions. *C*, cryo-EM structure of active NINJ1 oligomer (two subunits shown) (PDB code: 8UIP). Upon pyroptotic stimulation or ionic imbalance downstream of GSDM pore formation, the NINJ1 dimer dissociates and undergoes conformational remodeling. TM1 is refolded into two amphipathic helices, α1 and α2, exposing buried oligomerization interfaces. TM3 and TM4 form the filament core, whereas α1 and α2 mediate intersubunit packing. *D* and *E*, electrostatic surface representations of inactive NINJ1 dimer (*D*) and active NINJ1 oligomer (two subunits shown) (*E*). The surface of inactive NINJ1 is predominantly hydrophobic. Upon activation, the amphipathic helices α1 and α2 reconfigure the exterior surface, producing a filament with a hydrophilic convex side and a hydrophobic concave face. *F*, the active NINJ1 filament adopts a curved conformation because of kinks in TM3 and TM4. Multiple curved NINJ1 filaments close into ring-shaped assemblies that encircle and excise lipid bilayer fragments, releasing them as membrane discs and generating large lesions in the plasma membrane. *G*, cryo-EM structure of NINJ2 oligomers (PDB code: 8SZB). NINJ2 forms distorted filaments that arc inward and are unable to stably engage flat membrane surfaces, explaining their lack of membrane rupture–inducing activity. NINJ1 (Ninjurin 1), nerve injury–induced protein 1.

Human encodes two NINJ homologs, NINJ1 and NINJ2, which share 52% amino acid sequence identity and 67% similarity. Surprisingly, only NINJ1 exhibits membrane lytic activity (42). NINJ1 is a 16-kDa plasma membrane protein predicted to contain two TM helices (TM3 and TM4) and an amphipathic N-terminal helix exposed extracellularly (42) (Fig. 5*A*). It has been shown that NINJ1 predominantly exists as dimers or trimers in the resting state but transitions into large oligomeric assemblies ranging from 40 to 900 kDa upon activation (42, 136). Structural analysis of a less cytotoxic NINJ1 mutant (K45Q) revealed that the inactive NINJ1 stabilizes as a face-to-face symmetric homodimer embedded in the plasma membrane (137) (Fig. 5*B*). Each NINJ1 adopts a compact three-helix bundle by repositioning the predicted amphipathic N-terminal helix 1 (TM1) into the membrane. The two NINJ1 molecules in the dimer form extensive intramolecular interactions that shield the oligomerization interfaces for mature oligomeric assembly (137) (Fig. 5*B*). Upon pyroptotic stimuli or ionic imbalance initiated by GSDM pore formation, NINJ1 dissociates from its autoinhibited dimer and undergoes a dramatic structural rearrangement (136, 138, 139). The N-terminal amphipathic TM1 is reoriented and separated into two amphipathic helices, α1 and α2, causing the exposure of previously hidden oligomerization interfaces on TM1 and the other two TM helices. This transition enables NINJ1 to self-assemble into a filamentous array composed of four helices per subunit, arranged in an antiparallel configuration, with the original TM3 and TM4 serving as the core of the filament. Within this filament, helix α2 packs against the upper regions of TM3 and TM4 at the interface between adjacent subunits, whereas helix α1 spans across the lower-mid region of TM helices from a neighboring subunit (136, 138, 139). Interestingly, TM3 and TM4 are both kinked in the NINJ1 oligomer under such a structural arrangement, inducing curvature into the NINJ1 filament (Fig. 5*C*). Notably, the amphipathic nature of helices α1 and α2 converts the convex surface of the NINJ1 filament from hydrophobic to hydrophilic, whereas the concave side remains hydrophobic and interacts with membrane lipids (138, 139) (Fig. 5, *D* and *E*). When multiple curved NINJ1 filaments assemble into a close ring, its hydrophobic concave surface will effectively encircle and excise a lipid bilayer fragment, whereas the hydrophilic convex side will serve as a driving force to promote the release of these enclosed membrane discs, leaving holes in the membrane (138) (Fig. 5*F*). Consequently, at a certain threshold point, when cells no longer have sufficient material or capacity to repair these holes in the plasma membrane, membrane rupture occurs. The observation of free-floating membrane discs in the extracellular milieu of dying cells visualized by super-resolution microscopy provides strong visual evidence in support of this NINJ1-driven plasma membrane rupture model (138).

### Triggers for NINJ1 activation

As discussed above, NINJ1 drives plasma membrane rupture by assembling into membrane-embedded filaments that form large, discontinuous lesions in the membrane. However, the upstream signals, particularly the connection between GSDM pore formation and NINJ1 activation, have not been fully elucidated. A recent study proposes a two-step activation model for NINJ1 (140). In the first stage, NINJ1 assembles into membrane-embedded oligomers that form latent weak points in the plasma membrane without immediately compromising integrity. In the second stage, rapid osmotic imbalance and cellular swelling, which are caused by GSDM pore formation or other cellular events, induce mechanical tension and curvature stress on the plasma membrane. This mechanical perturbation may act as a physical cue that shifts the conformational equilibrium of NINJ1 from its inactive or low-activity state toward high-order oligomerization and filament formation, facilitating membrane rupture. Notably, pharmacological inhibition of osmotic swelling prevents plasma membrane rupture without affecting oligomer formation, demonstrating that mechanical tension, rather than oligomerization *per se*, serves as the critical trigger for membrane lesion opening (140). Echoing this mechanosensitive model, a recent study using a novel cellular-stretch system demonstrated that NINJ1 acts as a critical regulator of plasma membrane fragility under mechanical stress (141). The abundance of NINJ1 on the plasma membrane inversely correlated with the threshold force required to induce membrane rupture—cells with higher NINJ1 levels ruptured more readily under stretch, whereas NINJ1-deficient cells exhibited increased resistance to mechanical disruption. Importantly, this study reinforces the notion that in the context of pyroptosis, cellular swelling is not merely a passive consequence of osmotic imbalance but functions as a mechanical “fuse” that triggers NINJ1's membrane-shattering activity. The initiating signals that promote the first step of NINJ1 oligomerization are still under investigation. Recent studies have begun to shed light on potential upstream cues. For example, ATP stimulation of the purinergic P2X7 receptor triggers a robust influx of Ca^2+^, which in turn activates TMEM16F-mediated phospholipid scrambling (142). This lipid asymmetry disruption directly induces NINJ1-dependent cell lysis independently of canonical inflammasome components, such as pannexins or GSDMD. In addition, Ella *et al.* (140) demonstrated that ionomycin, a calcium ionophore, similarly promotes Ca^2+^-dependent NINJ1 oligomerization, further supporting the role of calcium signaling and membrane lipid remodeling in priming NINJ1 assembly. Collectively, these findings suggest a model in which NINJ1 activation is governed by a combination of biochemical triggers (*e.g.*, calcium influx, lipid scrambling) and biophysical forces (*e.g.*, membrane tension), converging to control its transition from a latent oligomeric state to a membrane-disruptive filament that executes plasma membrane rupture.

### Structural divergences between NINJ1 and NINJ2

NINJ1 and NINJ2 share significant sequence similarity but exhibit markedly distinct functional roles in lytic cell death. While NINJ1 functions as a critical executor of plasma membrane rupture, NINJ2 lacks such membrane-disrupting activity despite its structural resemblance (42). Recent cryo-EM studies revealed that both NINJ1 and NINJ2 can oligomerize into filamentous assemblies; however, their filament architectures differ significantly (139). NINJ1 assembles into relatively flat, rigid filaments capable of forming circular oligomers that encircle and excise portions of the plasma membrane, a process critical for membrane rupture. In contrast, NINJ2 forms distorted filaments that arc inward toward the cytoplasmic space and fail to complete the circular assemblies required for membrane scission (Fig. 5, *F* and *G*).

This functional disparity is driven in part by their distinct lipid interactions, particularly with cholesterol (139). In the NINJ2 filament structure, a well-resolved cholesterol molecule was observed. The bound cholesterol molecule induces a displacement of the TM4 helix and results in a distorted inverted trapezoid-shaped subunit. A critical determinant of this interaction is Gln103 in NINJ2, which forms additional hydrogen bonds with cholesterol, thereby enhancing cholesterol binding and driving the filament distortion. This distortion prevents the subunits in the NINJ2 filament from assembling into a flat membrane, thereby impairing membrane-disrupting activity. In contrast, the equivalent position in NINJ1 is occupied by a bulky hydrophobic phenylalanine (Phe117 in human NINJ1), which lacks hydrogen-bonding capability and does not promote such distortion-inducing interactions. As a result, NINJ1 maintains a more compact subunit architecture with kinked TM3 and TM4 helices that align into relatively flat filaments competent for circular assembly and membrane rupture (139) (Fig. 5, *F* and *G*). The functional relevance of this lipid interaction was further validated through cell-based assays, where mutating key residues involved in cholesterol coordination or modulating membrane lipid composition significantly altered filament curvature and impaired NINJ2’s ability to facilitate plasma membrane rupture (139).

Strikingly, a recent work demonstrated that replacing the N-terminal 30 amino acids of NINJ2 with the corresponding N-terminal region from NINJ1 was sufficient to confer the membrane rupture activity to NINJ2 (140). This finding highlights an unexpected role of the N-terminal region in enabling or regulating plasma membrane rupture of NINJs; however, the underlying mechanism remains unclear. Notably, the N termini of both NINJ1 and NINJ2 are disordered and are not directly involved in filament formation. Whether and how this N-terminal region contributes to modulating filament topology, stabilizing ring closure, or directly engaging lipid components will require further structural and biophysical investigation (140).

While current biochemical and structural studies suggest that NINJ2's inability to perforate membranes arises from its intrinsic property, we cannot exclude the possibility that additional regulatory factors, such as membrane-associated cofactors or PTMs, may modulate NINJ2 activity in specific cellular contexts. Further investigations are needed to elucidate whether NINJ2 can acquire membrane-rupturing capacity under certain physiological or pathological conditions. Collectively, these insights underscore that while NINJ1 and NINJ2 are structurally similar, subtle differences in lipid engagement, filament curvature, and N-terminal dynamics dictate their distinct functional specificities.

### Roles of NINJ1 beyond pyroptosis and its association with diseases

As the terminal executioner of plasma membrane rupture, NINJ1 has been implicated in multiple forms of lytic cell death beyond pyroptosis (143). In necroptosis, NINJ1 contributes to the final rupture step downstream of mixed lineage kinase domain–like protein activation (42). Although NINJ1 is not essential for initiating or executing necroptotic cell death, its deficiency has been shown to attenuate terminal plasma membrane rupture, as evidenced by reduced LDH release in BMDMs subjected to necroptotic stimuli, such as TNF combined with pan-caspase inhibition (42). In addition, NINJ1 is required for plasma membrane rupture during norovirus infection, where the virus encodes a mixed lineage kinase domain–like protein that induces cell death independently of receptor-interacting protein kinase 3 (144). In NINJ1-deficient cells, norovirus infection fails to promote LDH release, despite unaltered cell death caused by viral infection. NINJ1 also plays a role in secondary necrosis (or postapoptosis) (42, 145). In response to chemotherapeutic agents, such as cisplatin or venetoclax, apoptotic cells in NINJ1-deficient conditions exhibit characteristic ballooning but show impaired release of DAMPs, including HMGB1 and LDH, highlighting the role of NINJ1 in terminal plasma membrane rupture during secondary necrosis (42). Furthermore, recent studies suggest a potential involvement of NINJ1 in ferroptosis-associated plasma membrane rupture. While NINJ1 is dispensable for early ferroptotic events—including lipid peroxidation, ion channel–mediated calcium influx, and cellular swelling—its oligomerization coincides with the terminal loss of plasma membrane integrity during ferroptosis. NINJ1 deficiency partially protects macrophages and fibroblasts from ferroptosis-induced plasma membrane rupture (146). This observation raises the possibility that NINJ1 may act downstream of oxidative damage to amplify or facilitate membrane rupture, although the mechanistic connection between ferroptotic lipid peroxidation and NINJ1-mediated membrane disruption requires further investigation. Collectively, these findings support a model in which NINJ1 acts as an effector of plasma membrane rupture across diverse forms of lytic cell death, with its functional contribution varying depending on the specific cellular context and upstream death pathway.

NINJ1 also plays a unique role in ferroptosis that differs from its membrane-rupturing function. Instead of promoting plasma membrane rupture, NINJ1 regulates ferroptosis by controlling cellular metabolism (147). It was found that knocking down NINJ1 protects cancer cells from ferroptosis caused by class I ferroptosis inducers, such as xCT inhibitors. This protection comes from increased stability and activity of the xCT transporter, which boosts cystine uptake and raises levels of CoA and GSH, which are key molecules that defend against ferroptosis. Blocking CoA or GSH production removes this protective effect, whereas overexpressing NINJ1 reduces xCT levels and makes cells more sensitive to ferroptosis (147). Importantly, glycine, which prevents membrane rupture in pyroptosis, has no effect here, confirming that NINJ1 acts through a nonlytic mechanism (134, 147). These findings reveal a unique role of NINJ1 in cell death beyond membrane rupture.

NINJ1 is broadly expressed across various tissues, with particularly high expression in immune cells, such as macrophages and neutrophils, and in organs frequently affected by inflammatory diseases, including the liver, lungs, spleen, and brain. This expression profile aligns with its established role in lytic cell death and inflammation (143). NINJ1 has been implicated in several inflammatory and degenerative diseases. For instance, in models of pulmonary fibrosis and experimental autoimmune encephalomyelitis, Ninj1 deficiency significantly reduces tissue damage and inflammation, suggesting that NINJ1-mediated plasma membrane rupture exacerbates disease progression (148). Genome-wide association studies have also linked NINJ1 expression to serum liver enzyme levels, indicating a role in hepatocellular injury (149). NINJ1 is also co-opted by norovirus to promote viral egress through plasma membrane rupture, facilitating the release of both viral components and host DAMPs (150). Beyond inflammatory and infectious diseases, NINJ1 is found to be upregulated in various cancers, such as hepatocellular carcinoma, acute lymphoblastic B-cell leukemia, urothelial bladder cancer, and circulating prostate cancer, although its functional contribution to tumorigenesis remains to be elucidated. Collectively, these findings pinpoint NINJ1’s multifaceted role in host defense, pathological inflammation, and cancers, highlighting its potential as a therapeutic target for conditions involving dysregulated lytic cell death.

## Conclusion and future perspectives

Over the past decade, remarkable progress has been made in elucidating the molecular mechanisms underlying lytic cell death. The discovery and structural characterization of GSDMs as pore-forming executioners of pyroptosis have redefined our understanding of how cells disassemble under inflammatory stress. However, the realization that GSDM pore formation alone is insufficient to drive terminal plasma membrane rupture has prompted a broader search for additional effectors, culminating in the identification of NINJ1 as a conserved and essential mediator of plasma membrane rupture across multiple forms of lytic cell death (42, 144, 145, 146, 147, 151).

Despite major progress, several key questions remain, offering exciting opportunities for future investigation. For GSDMs, deciphering the full regulatory network remains a top priority. PTMs, such as ubiquitination, palmitoylation, PARylation, and phosphorylation, have emerged as critical modulators of GSDM activity (71, 93, 97, 98, 99, 100, 101, 103, 104, 105, 107, 108, 109, 110, 111, 125, 127). Understanding how these PTMs interact, compete, or synergize to modulate GSDM conformation, membrane targeting, and oligomerization will be essential for mapping the full regulatory network of GSDM activation. Integrating advanced mass spectrometry, proximity labeling, and structural proteomics may facilitate the discovery of novel PTMs and their regulatory enzymes. In parallel, the diversity of GSDM homologs across bacteria, fungi, and early diverging metazoans raises important evolutionary questions and reveals surprising mechanistic plasticity (30, 31, 34, 55, 92, 129, 130). Investigating these noncanonical activation modes may uncover conserved design principles and inform efforts to reprogram or engineer GSDMs for immunotherapy or targeted cytotoxicity. Moreover, recent findings that GSDM pores can propagate damage *via* EVs challenge the long-held view that pyroptosis is strictly cell autonomous (115). The potential for GSDM pores to mediate bystander killing, intercellular communication, or extracellular immune signaling opens new lines of inquiry regarding their physiological roles in immunity, autoimmunity, and cancer.

For NINJ1, future studies must clarify its precise activation mechanisms and regulatory checkpoints. Although GSDM-induced ionic imbalance and osmotic stress appear to be upstream triggers (140, 142), the mechanism by which these signals are sensed and transduced to initiate NINJ1’s conformational rearrangement and oligomerization remains poorly defined. While structural studies on NINJ1 and its homologs, NINJ2, and the proposed “cookie-cutter” model offer a compelling framework for NINJ1 activation, recent studies on the functions of the disordered N-terminal region in NINJ1 suggest a more intricate regulatory mechanism (136, 137, 138, 139, 140). In addition, emerging evidence indicates that NINJ1 may play nonlytic roles in regulating ferroptosis through modulation of xCT stability and CoA metabolism (147). These observations raise the intriguing possibility that NINJ1 acts as a broader modulator of cellular stress responses, redox balance, or metabolic homeostasis beyond its canonical role in membrane rupture.

From a translational perspective, both GSDMs and NINJ1 represent attractive therapeutic targets (47, 143, 152, 153). Inhibiting GSDM activation or blocking NINJ1 oligomerization could prevent excessive inflammation and tissue damage in diseases, such as sepsis, stroke, myocardial infarction, and autoimmune disorders (118, 149, 154, 155, 156). Conversely, therapeutic activation of these proteins might enhance antitumor immunity or promote clearance of infected or damaged cells in cases where immunological silence contributes to pathology (85, 86). The development of small-molecule inhibitors, peptidomimetics, or biologics targeting these pathways will be the next critical steps toward clinical translation.

In conclusion, the convergence of structural, biochemical, and functional insights has placed GSDMs and NINJ1 at the center of the lytic cell death research. However, it is important to recognize that this field is still in its infancy. The discoveries of GSDMs and NINJ1 as central executors of lytic cell death are relatively recent, and our current understanding is far from complete. Fundamental aspects such as the regulatory networks controlling GSDM activation, the precise molecular cues that trigger NINJ1 polymerization, and the functional interplay between these proteins in different cellular contexts remain incompletely elucidated. As research in this area accelerates, new structural and mechanistic paradigms are likely to emerge, filling these gaps and uncovering novel layers of regulation.

## Conflict of interest

The authors declare that they have no conflicts of interest with the contents of this article.

## References

[1] Galluzzi L., Vitale I., Aaronson S.A., Abrams J.M., Adam D., Agostinis P. (2018). Molecular mechanisms of cell death: recommendations of the nomenclature committee on cell death 2018. Cell Death Differ..

[2] Vitale I., Pietrocola F., Guilbaud E., Aaronson S.A., Abrams J.M., Adam D. (2023). Apoptotic cell death in disease-Current understanding of the NCCD 2023. Cell Death Differ..

[3] Tang D., Kang R., Berghe T.V., Vandenabeele P., Kroemer G. (2019). The molecular machinery of regulated cell death. Cell Res..

[4] Newton K., Strasser A., Kayagaki N., Dixit V.M. (2024). Cell death. Cell.

[5] Yuan J., Ofengeim D. (2024). A guide to cell death pathways. Nat. Rev. Mol. Cell Biol..

[6] Ellis H.M., Horvitz H.R. (1986). Genetic control of programmed cell death in the nematode C. elegans. Cell.

[7] Friedlander A.M. (1986). Macrophages are sensitive to anthrax lethal toxin through an acid-dependent process. J. Biol. Chem..

[8] Zychlinsky A., Prevost M.C., Sansonetti P.J. (1992). Shigella flexneri induces apoptosis in infected macrophages. Nature.

[9] Kerr J.F., Wyllie A.H., Currie A.R. (1972). Apoptosis: a basic biological phenomenon with wide-ranging implications in tissue kinetics. Br. J. Cancer.

[10] Chen Y., Smith M.R., Thirumalai K., Zychlinsky A. (1996). A bacterial invasin induces macrophage apoptosis by binding directly to ICE. EMBO J..

[11] Brennan M.A., Cookson B.T. (2000). Salmonella induces macrophage death by caspase-1-dependent necrosis. Mol. Microbiol..

[12] Hitomi J., Christofferson D.E., Ng A., Yao J., Degterev A., Xavier R.J. (2008). Identification of a molecular signaling network that regulates a cellular necrotic cell death pathway. Cell.

[13] Degterev A., Huang Z., Boyce M., Li Y., Jagtap P., Mizushima N. (2005). Chemical inhibitor of nonapoptotic cell death with therapeutic potential for ischemic brain injury. Nat. Chem. Biol..

[14] Degterev A., Hitomi J., Germscheid M., Ch'en I.L., Korkina O., Teng X. (2008). Identification of RIP1 kinase as a specific cellular target of necrostatins. Nat. Chem. Biol..

[15] Boise L.H., Collins C.M. (2001). Salmonella-induced cell death: apoptosis, necrosis or programmed cell death?. Trends Microbiol..

[16] Bergsbaken T., Fink S.L., Cookson B.T. (2009). Pyroptosis: host cell death and inflammation. Nat. Rev. Microbiol..

[17] Atabaki R., Khaleghzadeh-Ahangar H., Esmaeili N., Mohseni-Moghaddam P. (2023). Role of pyroptosis, a pro-inflammatory programmed cell death, in epilepsy. Cell Mol. Neurobiol..

[18] Laster S.M., Wood J.G., Gooding L.R. (1988). Tumor necrosis factor can induce both apoptic and necrotic forms of cell lysis. J. Immunol..

[19] Zhang Y., Chen X., Gueydan C., Han J. (2018). Plasma membrane changes during programmed cell deaths. Cell Res..

[20] Martinon F., Burns K., Tschopp J. (2002). The inflammasome: a molecular platform triggering activation of inflammatory caspases and processing of proIL-beta. Mol. Cell.

[21] Cho Y.S., Challa S., Moquin D., Genga R., Ray T.D., Guildford M. (2009). Phosphorylation-driven assembly of the RIP1-RIP3 complex regulates programmed necrosis and virus-induced inflammation. Cell.

[22] Zhao Y., Yang J., Shi J., Gong Y.N., Lu Q., Xu H. (2011). The NLRC4 inflammasome receptors for bacterial flagellin and type III secretion apparatus. Nature.

[23] Kanneganti T.D., Ozoren N., Body-Malapel M., Amer A., Park J.H., Franchi L. (2006). Bacterial RNA and small antiviral compounds activate caspase-1 through cryopyrin/Nalp3. Nature.

[24] Shi J., Zhao Y., Wang K., Shi X., Wang Y., Huang H. (2015). Cleavage of GSDMD by inflammatory caspases determines pyroptotic cell death. Nature.

[25] Kayagaki N., Stowe I.B., Lee B.L., O'Rourke K., Anderson K., Warming S. (2015). Caspase-11 cleaves gasdermin D for non-canonical inflammasome signalling. Nature.

[26] He W.T., Wan H., Hu L., Chen P., Wang X., Huang Z. (2015). Gasdermin D is an executor of pyroptosis and required for interleukin-1beta secretion. Cell Res..

[27] Shi J., Gao W., Shao F. (2017). Pyroptosis: gasdermin-mediated programmed necrotic cell death. Trends Biochem. Sci..

[28] Tamura M., Tanaka S., Fujii T., Aoki A., Komiyama H., Ezawa K. (2007). Members of a novel gene family, Gsdm, are expressed exclusively in the epithelium of the skin and gastrointestinal tract in a highly tissue-specific manner. Genomics.

[29] Katoh M., Katoh M. (2004). Identification and characterization of human DFNA5L, mouse Dfna5l, and rat Dfna5l genes in silico. Int. J. Oncol..

[30] Daskalov A., Gladieux P., Heller J., Glass N.L. (2019). Programmed cell death in Neurospora crassa is controlled by the allorecognition determinant rcd-1. Genetics.

[31] Johnson A.G., Wein T., Mayer M.L., Duncan-Lowey B., Yirmiya E., Oppenheimer-Shaanan Y. (2022). Bacterial gasdermins reveal an ancient mechanism of cell death. Science.

[32] Angosto-Bazarra D., Alarcon-Vila C., Hurtado-Navarro L., Banos M.C., Rivers-Auty J., Pelegrin P. (2022). Evolutionary analyses of the gasdermin family suggest conserved roles in infection response despite loss of pore-forming functionality. BMC Biol..

[33] Jiang S., Zhou Z., Sun Y., Zhang T., Sun L. (2020). Coral gasdermin triggers pyroptosis. Sci. Immunol..

[34] Daskalov A., Mitchell P.S., Sandstrom A., Vance R.E., Glass N.L. (2020). Molecular characterization of a fungal gasdermin-like protein. Proc. Natl. Acad. Sci. U. S. A..

[35] Liu X., Zhang Z., Ruan J., Pan Y., Magupalli V.G., Wu H. (2016). Inflammasome-activated gasdermin D causes pyroptosis by forming membrane pores. Nature.

[36] Ding J., Wang K., Liu W., She Y., Sun Q., Shi J. (2016). Pore-forming activity and structural autoinhibition of the gasdermin family. Nature.

[37] Ruan J.B., Xia S.Y., Liu X., Lieberman J., Wu H. (2018). Cryo-EM structure of the gasdermin A3 membrane pore. Nature.

[38] Aglietti R.A., Estevez A., Gupta A., Ramirez M.G., Liu P.S., Kayagaki N. (2016). GsdmD p30 elicited by caspase-11 during pyroptosis forms pores in membranes. Proc. Natl. Acad. Sci. U. S. A..

[39] Xia S., Zhang Z., Magupalli V.G., Pablo J.L., Dong Y., Vora S.M. (2021). Gasdermin D pore structure reveals preferential release of mature interleukin-1. Nature.

[40] Volchuk A., Ye A., Chi L., Steinberg B.E., Goldenberg N.M. (2020). Indirect regulation of HMGB1 release by gasdermin D. Nat. Commun..

[41] Kayagaki N., Dixit V.M. (2019). Rescue from a fiery death: a therapeutic endeavor. Science.

[42] Kayagaki N., Kornfeld O.S., Lee B.L., Stowe I.B., O'Rourke K., Li Q. (2021). NINJ1 mediates plasma membrane rupture during lytic cell death. Nature.

[43] Wang Y., Shao F. (2021). NINJ1, rupturing swollen membranes for cataclysmic cell lysis. Mol. Cell.

[44] Shi J., Zhao Y., Wang Y., Gao W., Ding J., Li P. (2014). Inflammatory caspases are innate immune receptors for intracellular LPS. Nature.

[45] Kayagaki N., Warming S., Lamkanfi M., Vande Walle L., Louie S., Dong J. (2011). Non-canonical inflammasome activation targets caspase-11. Nature.

[46] Broz P., Pelegrin P., Shao F. (2020). The gasdermins, a protein family executing cell death and inflammation. Nat. Rev. Immunol..

[47] Liu X., Xia S., Zhang Z., Wu H., Lieberman J. (2021). Channelling inflammation: gasdermins in physiology and disease. Nat. Rev. Drug Discov..

[48] Liu Y., Fang Y., Chen X., Wang Z., Liang X., Zhang T. (2020). Gasdermin E-mediated target cell pyroptosis by CAR T cells triggers cytokine release syndrome. Sci. Immunol..

[49] Kanneganti A., Malireddi R.K.S., Saavedra P.H.V., Vande Walle L., Van Gorp H., Kambara H. (2018). GSDMD is critical for autoinflammatory pathology in a mouse model of Familial Mediterranean Fever. J. Exp. Med..

[50] Doitsh G., Galloway N.L., Geng X., Yang Z., Monroe K.M., Zepeda O. (2014). Cell death by pyroptosis drives CD4 T-cell depletion in HIV-1 infection. Nature.

[51] Wang X., Wei X., Lu Y., Wang Q., Fu R., Wang Y. (2023). Characterization of GSDME in amphioxus provides insights into the functional evolution of GSDM-mediated pyroptosis. Plos Biol..

[52] Jaillon O., Aury J.M., Brunet F., Petit J.L., Stange-Thomann N., Mauceli E. (2004). Genome duplication in the teleost fish Tetraodon nigroviridis reveals the early vertebrate proto-karyotype. Nature.

[53] Wang C., Ruan J. (2023). An ancient defense mechanism: Conservation of gasdermin-mediated pyroptosis. Plos Biol..

[54] Billman Z.P., Kovacs S.B., Wei B., Kang K., Cisse O.H., Miao E.A. (2024). Caspase-1 activates gasdermin A in non-mammals. Elife.

[55] Clave C., Dyrka W., Turcotte E.A., Granger-Farbos A., Ibarlosa L., Pinson B. (2022). Fungal gasdermin-like proteins are controlled by proteolytic cleavage. Proc. Natl. Acad. Sci. U. S. A..

[56] Wang J., Deobald K., Re F. (2019). Gasdermin D protects from Melioidosis through pyroptosis and direct killing of bacteria. J. Immunol..

[57] Liu X., Lieberman J. (2020). Knocking 'em Dead: pore-forming proteins in immune defense. Annu. Rev. Immunol..

[58] Demarco B., Grayczyk J.P., Bjanes E., Le Roy D., Tonnus W., Assenmacher C.A. (2020). Caspase-8-dependent gasdermin D cleavage promotes antimicrobial defense but confers susceptibility to TNF-induced lethality. Sci. Adv..

[59] Schwarzer R., Jiao H., Wachsmuth L., Tresch A., Pasparakis M. (2020). FADD and caspase-8 regulate Gut homeostasis and inflammation by controlling MLKL- and GSDMD-mediated death of intestinal epithelial cells. Immunity.

[60] Kambara H., Liu F., Zhang X., Liu P., Bajrami B., Teng Y. (2018). Gasdermin D exerts anti-inflammatory effects by promoting neutrophil death. Cell Rep..

[61] Burgener S.S., Leborgne N.G.F., Snipas S.J., Salvesen G.S., Bird P.I., Benarafa C. (2019). Cathepsin G inhibition by Serpinb1 and Serpinb6 prevents programmed necrosis in neutrophils and Monocytes and reduces GSDMD-driven inflammation. Cell Rep..

[62] Sollberger G., Choidas A., Burn G.L., Habenberger P., Di Lucrezia R., Kordes S. (2018). Gasdermin D plays a vital role in the generation of neutrophil extracellular traps. Sci. Immunol..

[63] Deng W., Bai Y., Deng F., Pan Y., Mei S., Zheng Z. (2022). Streptococcal pyrogenic exotoxin B cleaves GSDMA and triggers pyroptosis. Nature.

[64] LaRock D.L., Johnson A.F., Wilde S., Sands J.S., Monteiro M.P., LaRock C.N. (2022). Group A Streptococcus induces GSDMA-dependent pyroptosis in keratinocytes. Nature.

[65] Rana N., Privitera G., Kondolf H.C., Bulek K., Lechuga S., De Salvo C. (2022). GSDMB is increased in IBD and regulates epithelial restitution/repair independent of pyroptosis. Cell.

[66] Sun Q., Yang J., Xing G., Sun Q., Zhang L., He F. (2008). Expression of GSDML associates with tumor progression in uterine Cervix cancer. Transl. Oncol..

[67] Das S., Miller M., Beppu A.K., Mueller J., McGeough M.D., Vuong C. (2016). GSDMB induces an asthma phenotype characterized by increased airway responsiveness and remodeling without lung inflammation. Proc. Natl. Acad. Sci. U. S. A..

[68] Cardamone G., Paraboschi E.M., Rimoldi V., Duga S., Solda G., Asselta R. (2017). The characterization of GSDMB splicing and Backsplicing profiles identifies novel isoforms and a circular RNA that are dysregulated in multiple sclerosis. Int. J. Mol. Sci..

[69] Morrison F.S., Locke J.M., Wood A.R., Tuke M., Pasko D., Murray A. (2013). The splice site variant rs11078928 may be associated with a genotype-dependent alteration in expression of GSDMB transcripts. BMC Genomics.

[70] Zhong X., Zeng H., Zhou Z., Su Y., Cheng H., Hou Y. (2023). Structural mechanisms for regulation of GSDMB pore-forming activity. Nature.

[71] Wang C., Shivcharan S., Tian T., Wright S., Ma D., Chang J. (2023). Structural basis for GSDMB pore formation and its targeting by IpaH7.8. Nature.

[72] Oltra S.S., Colomo S., Sin L., Perez-Lopez M., Lazaro S., Molina-Crespo A. (2023). Distinct GSDMB protein isoforms and protease cleavage processes differentially control pyroptotic cell death and mitochondrial damage in cancer cells. Cell Death Differ..

[73] Kong Q., Xia S., Pan X., Ye K., Li Z., Li H. (2023). Alternative splicing of GSDMB modulates killer lymphocyte-triggered pyroptosis. Sci. Immunol..

[74] Zhou Z., He H., Wang K., Shi X., Wang Y., Su Y. (2020). Granzyme A from cytotoxic lymphocytes cleaves GSDMB to trigger pyroptosis in target cells. Science.

[75] Panganiban R.A., Sun M., Dahlin A., Park H.R., Kan M., Himes B.E. (2018). A functional splice variant associated with decreased asthma risk abolishes the ability of gasdermin B to induce epithelial cell pyroptosis. J. Allergy Clin. Immunol..

[76] Ivanov A.I., Rana N., Privitera G., Pizarro T.T. (2023). The enigmatic roles of epithelial gasdermin B: recent discoveries and controversies. Trends Cell Biol..

[77] Miguchi M., Hinoi T., Shimomura M., Adachi T., Saito Y., Niitsu H. (2016). Gasdermin C is upregulated by inactivation of transforming growth factor beta receptor type II in the presence of mutated Apc, promoting colorectal cancer proliferation. PLoS One.

[78] Yang L., He H., Guo X.K., Wang J., Wang W., Li D. (2024). Intraepithelial mast cells drive gasdermin C-mediated type 2 immunity. Immunity.

[79] Hou J., Zhao R., Xia W., Chang C.W., You Y., Hsu J.M. (2020). PD-L1-mediated gasdermin C expression switches apoptosis to pyroptosis in cancer cells and facilitates tumour necrosis. Nat. Cell Biol..

[80] Zhang J.Y., Zhou B., Sun R.Y., Ai Y.L., Cheng K., Li F.N. (2021). The metabolite alpha-KG induces GSDMC-dependent pyroptosis through death receptor 6-activated caspase-8. Cell Res..

[81] Tan G., Huang C., Chen J., Chen B., Zhi F. (2021). Gasdermin-E-mediated pyroptosis participates in the pathogenesis of Crohn's disease by promoting intestinal inflammation. Cell Rep..

[82] Bischoff A.M., Luijendijk M.W., Huygen P.L., van Duijnhoven G., De Leenheer E.M., Oudesluijs G.G. (2004). A novel mutation identified in the DFNA5 gene in a Dutch family: a clinical and genetic evaluation. Audiol. Neurootol..

[83] Wang J., Ye T., Wang S., Wang J., Jin Y. (2022). Molecular mechanisms and therapeutic relevance of gasdermin E in human diseases. Cell Signal..

[84] Ye F., Zhang W., Fan C., Dong J., Peng M., Deng W. (2023). Antileukemic effect of venetoclax and hypomethylating agents via caspase-3/GSDME-mediated pyroptosis. J. Transl Med..

[85] Wang Y., Gao W., Shi X., Ding J., Liu W., He H. (2017). Chemotherapy drugs induce pyroptosis through caspase-3 cleavage of a gasdermin. Nature.

[86] Zhang Z., Zhang Y., Xia S., Kong Q., Li S., Liu X. (2020). Gasdermin E suppresses tumour growth by activating anti-tumour immunity. Nature.

[87] De Schutter E., Roelandt R., Riquet F.B., Van Camp G., Wullaert A., Vandenabeele P. (2021). Punching holes in cellular membranes: biology and evolution of gasdermins. Trends Cell Biol..

[88] Delmaghani S., del Castillo F.J., Michel V., Leibovici M., Aghaie A., Ron U. (2006). Mutations in the gene encoding pejvakin, a newly identified protein of the afferent auditory pathway, cause DFNB59 auditory neuropathy. Nat. Genet..

[89] Defourny J., Aghaie A., Perfettini I., Avan P., Delmaghani S., Petit C. (2019). Pejvakin-mediated pexophagy protects auditory hair cells against noise-induced damage. Proc. Natl. Acad. Sci. U. S. A..

[90] Liu Z., Wang C., Yang J., Zhou B., Yang R., Ramachandran R. (2019). Crystal structures of the full-length murine and human gasdermin D reveal mechanisms of autoinhibition, lipid binding, and oligomerization. Immunity.

[91] Chao K.L., Kulakova L., Herzberg O. (2017). Gene polymorphism linked to increased asthma and IBD risk alters gasdermin-B structure, a sulfatide and phosphoinositide binding protein. Proc. Natl. Acad. Sci. U. S. A..

[92] Li Y., Hou Y., Sun Q., Zeng H., Meng F., Tian X. (2024). Cleavage-independent activation of ancient eukaryotic gasdermins and structural mechanisms. Science.

[93] Du G., Healy L.B., David L., Walker C., El-Baba T.J., Lutomski C.A. (2024). ROS-dependent S-palmitoylation activates cleaved and intact gasdermin D. Nature.

[94] Sborgi L., Ruhl S., Mulvihill E., Pipercevic J., Heilig R., Stahlberg H. (2016). GSDMD membrane pore formation constitutes the mechanism of pyroptotic cell death. EMBO J..

[95] Mari S.A., Pluhackova K., Pipercevic J., Leipner M., Hiller S., Engel A. (2022). Gasdermin-A3 pore formation propagates along variable pathways. Nat. Commun..

[96] Mulvihill E., Sborgi L., Mari S.A., Pfreundschuh M., Hiller S., Muller D.J. (2018). Mechanism of membrane pore formation by human gasdermin-D. EMBO J..

[97] Hansen J.M., de Jong M.F., Wu Q., Zhang L.S., Heisler D.B., Alto L.T. (2021). Pathogenic ubiquitination of GSDMB inhibits NK cell bactericidal functions. Cell.

[98] Hu L., Chen M., Chen X., Zhao C., Fang Z., Wang H. (2020). Chemotherapy-induced pyroptosis is mediated by BAK/BAX-caspase-3-GSDME pathway and inhibited by 2-bromopalmitate. Cell Death Dis..

[99] Zhang N., Zhang J., Yang Y., Shan H., Hou S., Fang H. (2024). A palmitoylation-depalmitoylation relay spatiotemporally controls GSDMD activation in pyroptosis. Nat. Cell Biol..

[100] Balasubramanian A., Hsu A.Y., Ghimire L., Tahir M., Devant P., Fontana P. (2024). The palmitoylation of gasdermin D directs its membrane translocation and pore formation during pyroptosis. Sci. Immunol..

[101] Liu Z., Li S., Wang C., Vidmar K.J., Bracey S., Li L. (2024). Palmitoylation at a conserved cysteine residue facilitates gasdermin D-mediated pyroptosis and cytokine release. Proc. Natl. Acad. Sci. U. S. A..

[102] Rogers C., Erkes D.A., Nardone A., Aplin A.E., Fernandes-Alnemri T., Alnemri E.S. (2019). Gasdermin pores permeabilize mitochondria to augment caspase-3 activation during apoptosis and inflammasome activation. Nat. Commun..

[103] Santamaria A., Wang B., Elowe S., Malik R., Zhang F., Bauer M. (2011). The Plk1-dependent phosphoproteome of the early mitotic spindle. Mol. Cell Proteomics.

[104] Li Y., Pu D., Huang J., Zhang Y., Yin H. (2022). Protein phosphatase 1 regulates phosphorylation of gasdermin D and pyroptosis. Chem. Commun. (Camb).

[105] Li X., Li X., Xiang C., Cao J., Guo J., Zhu S. (2024). Starvation-induced phosphorylation activates gasdermin A to initiate pyroptosis. Cell Rep..

[106] Humphries F., Shmuel-Galia L., Ketelut-Carneiro N., Li S., Wang B., Nemmara V.V. (2020). Succination inactivates gasdermin D and blocks pyroptosis. Science.

[107] Chu X., Zhang T., Bukhari I., Hu M., Xu J., Xing Y. (2025). Ubiquitination of gasdermin D N-terminal domain directs its membrane translocation and pore formation during pyroptosis. Cell Death Dis..

[108] Luchetti G., Roncaioli J.L., Chavez R.A., Schubert A.F., Kofoed E.M., Reja R. (2021). Shigella ubiquitin ligase IpaH7.8 targets gasdermin D for degradation to prevent pyroptosis and enable infection. Cell Host Microbe.

[109] Shi Y., Yang Y., Xu W., Shi D., Xu W., Fu X. (2022). E3 ubiquitin ligase SYVN1 is a key positive regulator for GSDMD-mediated pyroptosis. Cell Death Dis..

[110] Li Y., Gao W., Qiu Y., Pan J., Guo Q., Liu X. (2025). RING1 dictates GSDMD-mediated inflammatory response and host susceptibility to pathogen infection. Cell Death Differ..

[111] Ren Y., Feng M., Hao X., Liu X., Li J., Li P. (2023). USP48 stabilizes gasdermin E to promote pyroptosis in cancer. Cancer Res..

[112] Devant P., Borsic E., Ngwa E.M., Xiao H., Chouchani E.T., Thiagarajah J.R. (2023). Gasdermin D pore-forming activity is redox-sensitive. Cell Rep..

[113] Wang Y., Shi P., Chen Q., Huang Z., Zou D., Zhang J. (2019). Mitochondrial ROS promote macrophage pyroptosis by inducing GSDMD oxidation. J. Mol. Cell Biol..

[114] Evavold C.L., Hafner-Bratkovic I., Devant P., D'Andrea J.M., Ngwa E.M., Borsic E. (2021). Control of gasdermin D oligomerization and pyroptosis by the Ragulator-Rag-mTORC1 pathway. Cell.

[115] Wright S.S., Kumari P., Fraile-Agreda V., Wang C., Shivcharan S., Kappelhoff S. (2025). Transplantation of gasdermin pores by extracellular vesicles propagates pyroptosis to bystander cells. Cell.

[116] Zeidman R., Jackson C.S., Magee A.I. (2009). Protein acyl thioesterases (Review). Mol. Membr. Biol..

[117] Margheritis E., Kappelhoff S., Danial J., Gehle N., Kohl W., Kurre R. (2024). Gasdermin D cysteine residues synergistically control its palmitoylation-mediated membrane targeting and assembly. EMBO J..

[118] Hu J.J., Liu X., Xia S., Zhang Z., Zhang Y., Zhao J. (2020). FDA-approved disulfiram inhibits pyroptosis by blocking gasdermin D pore formation. Nat. Immunol..

[119] Rathkey J.K., Zhao J., Liu Z., Chen Y., Yang J., Kondolf H.C. (2018). Chemical disruption of the pyroptotic pore-forming protein gasdermin D inhibits inflammatory cell death and sepsis. Sci. Immunol..

[120] Zhang N., Xu D. (2025). Controlling pyroptosis through post-translational modifications of gasdermin D. Dev. Cell.

[121] Yin H., Zheng J., He Q., Zhang X., Li X., Ma Y. (2023). Insights into the GSDMB-mediated cellular lysis and its targeting by IpaH7.8. Nat. Commun..

[122] Baker S., The H.C. (2018). Recent insights into Shigella. Curr. Opin. Infect. Dis..

[123] Lin J., Sun S., Zhao K., Gao F., Wang R., Li Q. (2023). Oncolytic Parapoxvirus induces Gasdermin E-mediated pyroptosis and activates antitumor immunity. Nat. Commun..

[124] Wei H., Yu X. (2016). Functions of PARylation in DNA damage repair pathways. Genomics Proteomics Bioinformatics.

[125] Zhou B., Jiang Z.H., Dai M.R., Ai Y.L., Xiao L., Zhong C.Q. (2024). Full-length GSDME mediates pyroptosis independent from cleavage. Nat. Cell Biol..

[126] Wu M., Wang Y., Yang D., Gong Y., Rao F., Liu R. (2019). A PLK1 kinase inhibitor enhances the chemosensitivity of cisplatin by inducing pyroptosis in oesophageal squamous cell carcinoma. EBioMedicine.

[127] Ai Y.L., Wang W.J., Liu F.J., Fang W., Chen H.Z., Wu L.Z. (2023). Mannose antagonizes GSDME-mediated pyroptosis through AMPK activated by metabolite GlcNAc-6P. Cell Res..

[128] Du G., Wu H. (2025). Gasdermin D pores hitch a ride: extracellular vesicles spread pyroptosis. Cell Res..

[129] Johnson A.G., Mayer M.L., Schaefer S.L., McNamara-Bordewick N.K., Hummer G., Kranzusch P.J. (2024). Structure and assembly of a bacterial gasdermin pore. Nature.

[130] Ren K., Farrell J.D., Li Y., Guo X., Xie R., Liu X. (2025). Mechanisms of RCD-1 pore formation and membrane bending. Nat. Commun..

[131] Tsuchiya K., Hosojima S., Hara H., Kushiyama H., Mahib M.R., Kinoshita T. (2021). Gasdermin D mediates the maturation and release of IL-1alpha downstream of inflammasomes. Cell Rep..

[132] Karmakar M., Minns M., Greenberg E.N., Diaz-Aponte J., Pestonjamasp K., Johnson J.L. (2020). N-GSDMD trafficking to neutrophil organelles facilitates IL-1beta release independently of plasma membrane pores and pyroptosis. Nat. Commun..

[133] Evavold C.L., Ruan J., Tan Y., Xia S., Wu H., Kagan J.C. (2018). The pore-forming protein gasdermin D regulates interleukin-1 secretion from living macrophages. Immunity.

[134] Borges J.P., Saetra R.S.R., Volchuk A., Bugge M., Devant P., Sporsheim B. (2022). Glycine inhibits NINJ1 membrane clustering to suppress plasma membrane rupture in cell death. Elife.

[135] Araki T., Milbrandt J. (1996). Ninjurin, a novel adhesion molecule, is induced by nerve injury and promotes axonal growth. Neuron.

[136] Degen M., Santos J.C., Pluhackova K., Cebrero G., Ramos S., Jankevicius G. (2023). Structural basis of NINJ1-mediated plasma membrane rupture in cell death. Nature.

[137] Pourmal S., Truong M.E., Johnson M.C., Yang Y., Zhou L., Alegre K. (2025). Autoinhibition of dimeric NINJ1 prevents plasma membrane rupture. Nature.

[138] David L., Borges J.P., Hollingsworth L.R., Volchuk A., Jansen I., Garlick E. (2024). NINJ1 mediates plasma membrane rupture by cutting and releasing membrane disks. Cell.

[139] Sahoo B., Mou Z., Liu W., Dubyak G., Dai X. (2025). How NINJ1 mediates plasma membrane rupture and why NINJ2 cannot. Cell.

[140] Ella Hartenian E.M.B., Ammirati G., Monnier S., Leloup H.B., Magalie Agustoni L.B., Carlos Santos J. (2024). Ninjurin-1 mediated plasma membrane rupture is a two-step process requiring cell swelling. bioRxiv.

[141] Zhu Y., Xiao F., Wang Y., Wang Y., Li J., Zhong D. (2025). NINJ1 regulates plasma membrane fragility under mechanical strain. Nature.

[142] Jazlyn P., Borges Y.W., Liron D., Volchuk A., Martins B., Ruiqi (2025). NINJ1 is activated by calcium-driven plasma membrane lipid scrambling during lytic cell death. bioRxiv.

[143] Hur C.J., Steinberg B.E. (2025). Targeting NINJ1-mediated cell rupture to treat inflammatory diseases. Mol. Med..

[144] Wang G., Zhang D., Orchard R.C., Hancks D.C., Reese T.A. (2023). Norovirus MLKL-like protein initiates cell death to induce viral egress. Nature.

[145] Dondelinger Y., Priem D., Huyghe J., Delanghe T., Vandenabeele P., Bertrand M.J.M. (2023). NINJ1 is activated by cell swelling to regulate plasma membrane permeabilization during regulated necrosis. Cell Death Dis..

[146] Ramos S., Hartenian E., Santos J.C., Walch P., Broz P. (2024). NINJ1 induces plasma membrane rupture and release of damage-associated molecular pattern molecules during ferroptosis. EMBO J..

[147] Chen S.Y., Wu J., Chen Y., Wang Y.E., Setayeshpour Y., Federico C. (2024). NINJ1 regulates ferroptosis via xCT antiporter interaction and CoA modulation. Cell Death Dis..

[148] Choi S., Woo J.K., Jang Y.S., Kang J.H., Hwang J.I., Seong J.K. (2018). Ninjurin1 plays a crucial role in pulmonary fibrosis by promoting interaction between macrophages and Alveolar epithelial cells. Sci. Rep..

[149] Kayagaki N., Stowe I.B., Alegre K., Deshpande I., Wu S., Lin Z. (2023). Inhibiting membrane rupture with NINJ1 antibodies limits tissue injury. Nature.

[150] Song J., Zhang L., Moon S., Fang A., Wang G., Gheshm N. (2025). Norovirus co-opts NINJ1 for selective protein secretion. Sci. Adv..

[151] Han J.H., Karki R., Malireddi R.K.S., Mall R., Sarkar R., Sharma B.R. (2024). NINJ1 mediates inflammatory cell death, PANoptosis, and lethality during infection conditions and heat stress. Nat. Commun..

[152] Kayagaki N., Webster J.D., Newton K. (2024). Control of cell death in health and disease. Annu. Rev. Pathol..

[153] Bai Y., Pan Y., Liu X. (2025). Mechanistic insights into gasdermin-mediated pyroptosis. Nat. Rev. Mol. Cell. Biol..

[154] Zhuang L., Luo X., Wu S., Lin Z., Zhang Y., Zhai Z. (2022). Disulfiram alleviates pristane-induced lupus via inhibiting GSDMD-mediated pyroptosis. Cell Death Discov..

[155] Silva C.M.S., Wanderley C.W.S., Veras F.P., Sonego F., Nascimento D.C., Goncalves A.V. (2021). Gasdermin D inhibition prevents multiple organ dysfunction during sepsis by blocking NET formation. Blood.

[156] Olsen M.B., Gregersen I., Sandanger O., Yang K., Sokolova M., Halvorsen B.E. (2022). Targeting the inflammasome in cardiovascular disease. JACC Basic Transl. Sci..

